# ChatGPT Responses in Hard Flaccid Syndrome: Exploratory Evaluation

**DOI:** 10.2196/93849

**Published:** 2026-11-10

**Authors:** Maher Abdessater, Ramy Touma Sawaya, Oussama Nasrallah, Samer Traboulsi, Bassel Bachir

**Affiliations:** 1 American University of Beirut Medical Center, P.O. Box 11-0236, Beirut, Beyrouth, 1107 2020, Lebanon, 961 01350000 ext 5913; 2 University Medical Center Rizk Hospital, Beirut, Beyrouth, Lebanon

**Keywords:** hard flaccid syndrome, artificial intelligence, AI, ChatGPT, rare andrological disorders, medical information accuracy

## Abstract

**Background:**

Hard flaccid syndrome (HFS) is a rare and poorly described andrological condition characterized by a persistent semirigid penis in the flaccid state and frequently accompanied by erectile dysfunction, sensory disturbances, urinary symptoms, and psychosocial distress. Due to the absence of standardized diagnostic and therapeutic guidelines, patients increasingly seek health information from AI-based tools such as ChatGPT. However, the reliability of such tools in rare diseases remains uncertain.

**Objective:**

This study aimed to perform an exploratory evaluation of the accuracy, readability, and short-term reproducibility of ChatGPT responses to patient-generated questions regarding HFS.

**Methods:**

Sixteen clinically relevant questions spanning 4 domains (epidemiology, treatment, diagnostics, and symptoms and case presentation) were developed using authentic patient-derived queries and previously published AI evaluations in rare diseases. Questions were submitted to the ChatGPT web interface (OpenAI) in separate chat sessions and repeated after 48 hours to assess short-term reproducibility. Three urologists with expertise in male sexual health independently evaluated response accuracy using a validated 4-point scale. Readability was assessed using the Gunning Fog Index, whereas interrater reliability was evaluated using intraclass correlation coefficients (ICCs).

**Results:**

Overall readability remained stable between sessions, with mean Gunning Fog Index scores of 15.19 (SD 1.68) at baseline and 14.59 (SD 1.66) after 48 hours (*P*=.33), corresponding to an upper high school to early college reading level. No significant differences in readability were observed across domains at either time point (T1 [November 27, 2025]: *P*=.76; T2 [December 1, 2025]: *P*=.68). Accuracy differed across clinical domains. At T1, a total of 43.8% (7/16) of the responses were classified as correct and comprehensive, 50% (8/16) were classified as correct but inadequate, and 6.3% (1/16) were classified as having mixed correct and incorrect information; no responses were classified as completely incorrect. At T2, performance improved, with 62.5% (10/16) of responses classified as correct and comprehensive and 37.5% (6/16) classified as correct but inadequate. Interrater reliability demonstrated fair agreement at the individual reviewer level and moderate agreement when ratings were averaged (T1 single-measure ICC=0.36, 95% CI 0.06‐0.67; T1 average-measure ICC=0.63, 95% CI 0.17‐0.86; T2 single-measure ICC=0.33, 95% CI 0.04‐0.64; T2 average-measure ICC=0.59, 95% CI 0.10‐0.84). Short-term reproducibility was substantial, with 87.5% (14/16) of responses receiving identical final accuracy classifications between sessions.

**Conclusions:**

ChatGPT demonstrated stable readability and high short-term consistency when addressing HFS-related questions. However, accuracy remained variable across clinical domains, with responses occasionally extending beyond the available evidence base in this poorly characterized condition. These findings suggest that AI tools may serve as accessible informational resources but should complement rather than replace expert clinical evaluation and evidence-based guidance, particularly in rare diseases for which clinical consensus remains limited.

## Introduction

Hard flaccid syndrome (HFS) is a rare, acquired disorder that manifests as a semirigid penis when flaccid without stimulation with erectile dysfunction; perineal and penile sensory changes such as burning, coldness, numbness, and pain; urinary symptoms such as painful urination and weak flow; and emotional and psychosocial distress [1]. While the etiology and pathophysiology are still unclear, some studies propose a relation with activation of the pelvic or pudendal hypogastric reflux [2] or past trauma to the associated neurovasculature [3,4].

As evidence is still scarce regarding HFS, treatment options are still limited to conservative management that targets improving psychosocial triggers, pelvic floor muscle relaxation, analgesia, and phosphodiesterase 5 inhibitors for erectile dysfunction [5-7]. These treatment options, while individually beneficial, have not yet been established as an effective standardized guideline for HFS.

As AI becomes increasingly integrated into everyday life, health care has undergone significant transformation, giving patients faster and more efficient access to medical information, diagnostic tools, and treatment options [8,9]. Despite these advancements, significant concerns remain about incorporating AI into health care as errors, limited training data, and hallucinated outputs are still common—particularly in the context of rare conditions [10-13].

Given the rarity of HFS, the limited evidence base, and the exploratory nature of evaluating AI performance in this setting, this study was designed as an exploratory assessment intended to characterize preliminary patterns in the accuracy, readability, and reproducibility of AI-generated information rather than provide definitive performance estimates. As such, this study aims to assess the accuracy, readability, and short-term reproducibility of AI model responses to common questions regarding HFS epidemiology, symptoms and risk factors, diagnostics, and therapy options.

## Methods

### Input Questions

This exploratory study evaluated the performance of ChatGPT (OpenAI) in addressing patient-generated questions related to HFS. The study questions were developed using authentic patient queries encountered in clinical practice supplemented by questions reported in prior investigations of rare diseases unrelated to HFS [14,15]. This study focused on questions with common use and broad clinical relevance, leaving out any that were nonclinical in nature, insufficiently precise, or tailored to a single patient’s situation.

Responses were generated using the publicly available ChatGPT web interface accessed through a free user account. Sixteen predefined questions were independently submitted as separate prompts in newly initiated chat sessions to minimize contextual carryover between responses. Responses were not regenerated, edited, or refined after their initial generation. The same prompting procedure was repeated 48 hours later to assess short-term reproducibility.

No API access, fine-tuning, or external model modifications were used. Data collection was performed between November 27, 2025 (T1), and December 1, 2025 (T2). No custom instructions were used. Memory settings were not intentionally modified and were left at the account’s default configuration during data collection. Web search functionality was not intentionally invoked during any prompting session. The domains and exact prompts are shown in Table 1. The responses at T1 and T2, copied and pasted directly from the ChatGPT chat, are shown in Multimedia Appendices 1 and 2 respectively.

**Table 1.** Domains and questions submitted to ChatGPT.

Domains	Questions
Epidemiology	How rare is HFS and how many people suffer from it worldwide? What are the risk factors or predispositions that can cause HFS? What patient characteristics are commonly associated with developing HFS? How many people might have HFS without knowing?
Treatment	What treatment options are available for HFS? Are there any preventative methods to avoid HFS? What research is being done to treat HFS? Are the available treatment options for HFS curative or just preventative?
Diagnostics and related concerns	What are the available diagnostic modalities for HFS? How can I differentiate HFS from other similar conditions? What conditions are similar to HFS and what are other potential differential diagnoses? Has anyone experienced worsening symptoms or new problems after trying treatments for HFS?
Symptoms and case presentation	Does HFS affect my sexual and reproductive health? What is the cause of the symptoms of HFS? Are there long-term side effects of HFS? Are the symptoms of HFS dangerous? Can they be a sign of something worrisome?

### Response Grading

Every answer was graded independently by 3 English-speaking urologists with expertise in male sexual health. Any discrepancies in grading were further assessed by a blinded fourth urologist. For reporting purposes, a single final consensus accuracy score was assigned to each response after adjudication and was subsequently used for all descriptive analyses, figures, and reproducibility assessments.

Readability of the text responses was assessed using the Gunning Fog Index (GFI), which is a validated scoring system that checks for wordiness and use of complex wording [16].

To assess accuracy, each grader independently analyzed the response thoroughly using a scoring system adapted from the work by Yeo et al [15], classifying responses as correct and comprehensive (4), correct but inadequate (3), having mixed correct and incorrect information (2), or completely incorrect (1). The interrater reliability (IRR) of these ordinal accuracy assessments was subsequently evaluated using the intraclass correlation coefficient (ICC), as described in the Statistical Analysis section. Higher accuracy scores indicated better performance, with grade 4 representing correct and comprehensive responses and grade 1 representing completely incorrect responses. The grades given by each individual reviewer are shown in Multimedia Appendix 3.

Short-term reproducibility was assessed by comparing the final adjudicated accuracy classifications assigned to responses generated at T1 and T2 using identical prompts. Reproducibility was defined as identical final accuracy ratings across both evaluation sessions.

### Statistical Analysis

Descriptive statistics were calculated for all outcomes within each domain. Given the small sample size within each domain (n=4 questions), formal normality testing was not performed as such tests have limited interpretability in very small samples. Descriptive statistics are presented as means and SDs. Paired Student independent 2-tailed *t* tests were used to compare overall readability scores between T1 and T2 across all 16 questions, whereas nonparametric methods were selected a priori for comparisons across domains.

IRR among the 3 independent reviewers was assessed using the ICC. The ICC was selected over κ-based statistics because more than 2 raters were involved and ratings were assigned on a 4-point ordinal scale, for which ICC is commonly used to estimate agreement while accounting for both systematic and random sources of variance. Reliability analyses were performed separately for T1 and T2 using a 2-way random-effects model with absolute agreement assuming that both raters and evaluated items constituted random samples from larger populations.

To examine differences in readability across domains within each evaluation session, the Kruskal-Wallis test was applied. This nonparametric test was chosen due to the small sample size and to avoid assumptions of normality and homogeneity of variance.

The 95% CIs were reported where applicable, and statistical significance was defined as a *P* value below .05. All analyses were conducted using SPSS Statistics for Windows (version 28.0; IBM Corp). No procedures for handling missing or imbalanced data were required as the analysis was based on complete textual prompts and AI-generated outputs rather than conventional structured datasets. All outputs were generated using the ChatGPT web interface using GPT-4.1, which was available at the time of data collection. No API access, fine-tuning, custom instructions, retrieval augmentation, or model parameter modifications were applied.

### Ethical Considerations

This study did not involve human participants, patient recruitment, patient records, identifiable personal information, or any intervention involving living individuals. All analyses were performed exclusively on AI-generated responses to predefined, non–patient-specific prompts submitted by the investigators. Consequently, this work does not constitute human participant research and did not require institutional review board approval or informed consent according to institutional and international research ethics principles governing studies that do not involve human participants or identifiable data. No patient-derived data were collected, stored, or analyzed at any stage of the study.

As this study represents a methodological evaluation of publicly accessible AI outputs rather than an observational or interventional human research study, no formal ethics application was submitted, and no protocol preregistration was required.

## Results

### Readability of ChatGPT Responses

Across all 16 questions related to HFS, the mean GFI score showed a small numerical decrease from 15.19 (SD 1.68) at T1 to 14.59 (SD 1.66) at T2, indicating largely stable linguistic complexity between the 2 evaluation sessions. Overall readability remained at a level consistent with upper high school to early college comprehension. Mean GFI scores by domain and overall at both evaluation sessions are summarized in Table 2.

When stratified by domain, mean GFI scores at T1 ranged from 14.55 (SD 2.36) to 15.70 (SD 0.91), with the lowest readability complexity observed in the treatment domain. At T2, mean GFI scores ranged from 13.81 (SD 0.85) to 15.23 (SD 2.83), with the greatest variability observed in the symptoms and case presentation domain.

**Table 2.** Readability of ChatGPT responses assessed using the Gunning Fog Index (GFI). GFI scores estimate text readability, with higher scores indicating greater linguistic complexity. T1 (November 27, 2025) and T2 (December 1, 2025) represent responses generated 48 hours apart using identical prompts.

Domain	GFI at T1, mean (SD; range)	GFI at T2, mean (SD; range)
Epidemiology	15.04 (1.57; 12.98‐16.68)	13.81 (0.85; 12.71‐14.75)
Treatment	14.55 (2.36; 12.65‐17.89)	14.68 (1.79; 12.26‐16.31)
Diagnostics	15.46 (2.06; 13.20‐17.32)	14.64 (0.72; 13.92‐15.48)
Symptoms and case presentation	15.70 (0.91; 14.74‐16.52)	15.23 (2.83; 12.83‐19.11)
Overall	15.19 (1.68; 12.65‐17.89)	14.59 (1.66; 12.26‐19.11)

### Comparison of Readability Between Evaluation Sessions

Paired analysis comparing readability scores between evaluation sessions demonstrated a small, nonsignificant numerical decrease in GFI from T1 to T2. The mean paired difference (GFIT1 − GFIT2) was 0.60 (95% CI −0.67 to 1.86). This difference did not reach statistical significance (*t*_15_=1.01; 2-sided *P*=.33). The associated effect size was small (Cohen *d*=0.25, 95% CI −0.25 to 0.75), indicating minimal practical difference in linguistic complexity between responses generated at the 2 time points. These findings suggest that overall readability of ChatGPT responses related to HFS remained stable across evaluation sessions.

### Readability Differences Across Domains

Nonparametric comparison of readability across the 4 predefined content domains was performed using the Kruskal-Wallis test. At the first evaluation session (T1), no statistically significant differences in GFI scores were observed between domains (Kruskal-Wallis *H*_3_=1.17; *P*=.76) despite modest variation in mean ranks across domains. Similarly, at the second evaluation session (T2), GFI scores did not differ significantly by domain (*H*_3_=1.52; *P*=.68). These findings indicate that the linguistic complexity of ChatGPT-generated responses related to HFS was broadly consistent across the epidemiology, treatment, diagnostics, and symptoms and case presentation domains at both time points.

### Accuracy and IRR

The distribution of accuracy grades varied across domains and evaluation sessions, as shown in Table 3. At T1, a total of 43.8% (7/16) of the responses were classified as correct and comprehensive (grade 4), 50% (8/16) were classified as correct but inadequate (grade 3), and 6.3% (1/16) were classified as having mixed correct and incorrect information (grade 2). No responses were classified as completely incorrect (grade 1).

At T2, performance improved further, with 62.5% (10/16) of responses classified as correct and comprehensive and 37.5% (6/16) classified as correct but inadequate. No responses received grades of 1 or 2.

**Table 3.** Distribution of accuracy grades assigned to ChatGPT responses across domains and evaluation sessions (N=16 per evaluation session).

Domain	T1^a^ score, n (%)				T2^b^ score, n (%)			
	4	3	2	1	4	3	2	1
Epidemiology	2 (12.5)	2 (12.5)	0 (0)	0 (0)	3 (18.8)	1 (6.3)	0 (0)	0 (0)
Treatment	3 (18.8)	1 (6.3)	0 (0)	0 (0)	3 (18.8)	1 (6.3)	0 (0)	0 (0)
Diagnostics	2 (12.5)	1 (6.3)	1 (6.3)	0 (0)	3 (18.8)	1 (6.3)	0 (0)	0 (0)
Symptoms and case presentation	0 (0)	4 (25)	0 (0)	0 (0)	1 (6.3)	3 (18.8)	0 (0)	0 (0)
Overall	7 (43.8)	8 (50)	1 (6.3)	0 (0)	10 (62.5)	6 (37.5)	0 (0)	0 (0)

a T1: November 27, 2025.

b T2: December 1, 2025.

Performance varied according to clinical domain. Epidemiology, treatment, and diagnostics demonstrated predominantly grade 4 classifications at both evaluation sessions, whereas responses related to symptoms and case presentation were more frequently classified as grade 3 (correct but inadequate), suggesting greater difficulty in addressing nuanced symptom interpretation and patient counseling questions. Overall, these findings suggest that ChatGPT performed better when providing general descriptive information than when addressing more complex clinical topics requiring nuanced evidence synthesis and interpretation. The analyses presented below were based on the final adjudicated accuracy score assigned to each response following reviewer consensus.

At T1, IRR demonstrated fair agreement at the level of individual reviewers, with a single-measure ICC of 0.36 (95% CI 0.06‐0.67, *P*<.01). Agreement improved to a moderate level when ratings were averaged across reviewers, with an average-measure ICC of 0.63 (95% CI 0.17‐0.86, *P*<.01). At T2, IRR remained statistically significant and showed comparable agreement. The single-measure ICC was 0.33 (95% CI 0.04‐0.64, *P*=.01), indicating fair agreement between individual reviewers, whereas the average-measure ICC reached 0.59 (95% CI 0.10‐0.84, *P*=.01), reflecting moderate agreement when reviewer scores were averaged. All ICC estimates were derived using a 2-way random-effects model with absolute agreement. Overall, the IRR of accuracy assessments was fair at the individual reviewer level and moderate when ratings were averaged across reviewers at both evaluation sessions. Internal consistency among reviewers was acceptable at T1 and moderate at T2, as summarized in Table 4. The distribution of accuracy scores for individual questions across domains and evaluation sessions is illustrated in Figure 1.

**Table 4.** Interrater reliability (IRR) among 3 reviewers.^a^

Time point and measure	ICC^b^ (95% CI)	*P* value	Interpretation
T1			
Single measures	0.36 (0.06‐0.67)	<.001	Fair agreement
Average measures	0.63 (0.17‐0.86)	<.001	Moderate agreement
T2			
Single measures	0.33 (0.04‐0.64)	.01	Fair agreement
Average measures	0.59 (0.10‐0.84)	.01	Moderate agreement

a Intraclass correlation coefficients were calculated using a 2-way random-effects model with absolute agreement. Higher intraclass correlation coefficient values indicate greater IRR. Internal consistency among reviewers was acceptable at T1 (November 27, 2025; Cronbach α=0.74) and moderate at T2 (December 1, 2025; Cronbach α=0.61).

b ICC: intraclass correlation coefficient.

**Figure 1.**
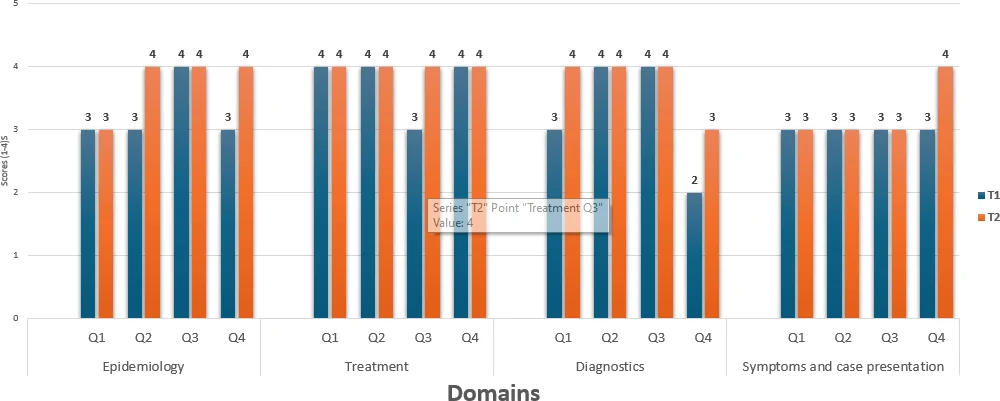
Distribution of final adjudicated accuracy scores across domains at T1 (November 27, 2025) and T2 (December 1, 2025). The scores shown represent the single final accuracy classification assigned to each response following independent evaluation by 3 expert urologists and adjudication of discrepancies by a fourth blinded reviewer. T1 and T2 responses were generated using identical prompts. Accuracy was graded using a 4-point scale: correct and comprehensive (4), correct but inadequate (3), mixed correct and incorrect information (2), and completely incorrect (1). Higher scores indicate better performance. Q: question.

### Reproducibility of Responses

Overall reproducibility of ChatGPT responses between the 2 evaluation sessions was high. Overall accuracy classification stability was high, with 87.5% (14/16) of responses receiving identical final accuracy ratings between T1 and T2. When stratified by domain, reproducibility was 100% in the epidemiology and symptoms and case presentation domains. In the treatment and diagnostics domains, 1 response in each domain demonstrated a change in reproducibility classification between sessions, resulting in a reproducibility rate of 75% for both domains. These findings indicate high short-term consistency in the reproducibility of ChatGPT-generated responses related to HFS across evaluation sessions for most patient-generated questions. Reproducibility outcomes across domains are summarized in Table 5.

**Table 5.** Reproducibility of ChatGPT responses between evaluation sessions. Accuracy classification stability was defined as identical final accuracy ratings assigned to responses generated at T1 (November 27, 2025) and T2 (December 1, 2025).

Domain	Questions, n	Reproducible responses, n/N (%)
Epidemiology	4	4/4 (100)
Treatment	4	3/4 (75)
Diagnostics	4	3/4 (75)
Symptoms and case presentation	4	4/4 (100)
Overall	16	14/16 (87.5)

## Discussion

### Principal Findings

This study provides one of the first structured evaluations of a large language model’s (LLM) performance in addressing a rare and poorly characterized andrological condition—HFS. Given the scarcity of high-quality clinical guidance and patients’ increasing reliance on online and AI-generated health information, assessing the accuracy, readability, and reproducibility of ChatGPT responses in this context is both timely and clinically relevant. Overall, ChatGPT demonstrated stable readability and high short-term reproducibility, whereas accuracy varied across clinical domains, supporting its potential as an informational resource but highlighting important limitations in rare diseases.

Performance varied across domains. Epidemiology, treatment, and diagnostics more frequently achieved correct and comprehensive responses, whereas symptoms and case presentation responses were more commonly classified as correct but inadequate, suggesting greater difficulty in addressing nuanced symptom interpretation and patient counseling questions. This pattern aligns with existing meta-analytic evidence on LLM performance: a systematic review and meta-analysis reported moderate accuracy (approximately 56%) of ChatGPT across medical query domains but emphasized significant heterogeneity and lack of standardized evaluation protocols [17]. Other quantitative assessments of LLM medical capabilities show similar constraints: LLMs can perform reasonably in examination-style settings but exhibit hallucinations, incorrect inferences, and poor sourcing outside structured prompts or training data [18]. These limitations are particularly concerning in rare disease contexts where corroborating literature is minimal, and patient decisions rely disproportionately on explanation and context.

Considering the clinical ambiguity surrounding HFS and its rarity, there is marked uncertainty in medical grading of model outputs as what may be labeled accurate by an AI tool could reflect prevailing beliefs, anecdotes, or conjectures rather than evidence. This highlights a central challenge for LLM evaluation in rare conditions where gold-standard clinical guidance does not yet exist.

It is critical to emphasize that model accuracy must be interpreted relative to the evidence base itself. For conditions such as HFS with evolving clinical descriptions and few high-quality studies, a model could correctly summarize what is written in the literature but still provide information with limited clinical utility because the underlying literature is limited.

One strong finding of our study was the high short-term reproducibility observed across repeated prompts. This stability is consistent with research showing that modern LLM outputs can be remarkably consistent in controlled settings when the prompt and model version are held constant [19]. However, reproducibility alone does not equate to factual correctness. A model that consistently reproduces incorrect reasoning or unvalidated mechanisms provides false reassurance rather than reliable guidance. This distinction reinforces that stability in outputs is a necessary but insufficient quality for medical use. Importantly, reproducibility and accuracy classification stability were evaluated as separate outcomes. Reproducibility in this study reflected the stability of expert-assigned accuracy classifications across repeated prompts rather than an assessment of semantic similarity between paired responses.

LLM outputs tended to be linguistically coherent and structured, which may increase perceived credibility among lay users. Prior work on health information retrieval has shown that the readability and perceived coherence of a source, whether AI or human, can influence perceived trustworthiness and intention to act even when the content is inaccurate [20]. Such effects raise ethical concerns when disseminating medical advice in high-anxiety contexts such as sexual dysfunction.

Beyond the findings of the present study, an important concern consistently highlighted in the literature is AI hallucinations, wherein generative models produce plausible but erroneous or fabricated content. Surveys of hallucination research highlight that even advanced models continue to generate inaccurate details and invented assertions absent direct evidence [21]. Hallucinations are strongly observed when models encounter low-data or ambiguous domains, as is the case with HFS. In addition, empirical work outside of health care contexts shows that LLMs often prioritize fluent agreement over factual accuracy when presented with illogical or poorly defined questions [22]. These intrinsic limitations underscore the caution that medical professionals and educators have expressed in deploying chatbot responses directly as medical advice, particularly when sources cannot be traced back to validated clinical evidence.

A qualitative review of several responses further illustrates these limitations. For example, when asked about the global prevalence of HFS, ChatGPT occasionally provided speculative statements regarding the number of affected individuals despite the absence of robust epidemiological data in the literature. Similarly, when discussing available treatment options, some responses implied a greater degree of therapeutic consensus than currently exists despite the absence of standardized management guidelines. Although these statements were often accompanied by appropriate caveats regarding limited evidence, they may nevertheless convey an unwarranted sense of certainty to patients seeking information about a poorly characterized condition. These examples underscore how LLMs may generate plausible and coherent narratives that extend beyond the available evidence base.

Several limitations should be acknowledged when interpreting these findings. First, the evaluation was limited to a single LLM and a single model version at a specific point in time; as such, the results may not be generalizable to other AI systems or to future iterations that may incorporate improved medical grounding or retrieval mechanisms. Second, although expert review was used as the reference standard for accuracy, the absence of consensus definitions, validated diagnostic criteria, and high-quality therapeutic evidence on HFS inherently limits the objectivity of accuracy assessments and likely contributed to only fair to moderate IRR. Third, the limited number of questions (16 in total; 4 per domain) reflects the exploratory nature of this study and may not fully capture the breadth, complexity, or heterogeneity of patient concerns related to HFS. Consequently, the findings should be interpreted as preliminary and hypothesis generating rather than definitive estimates of AI performance. Additionally, certain patient-generated questions may overlap across clinical domains, and categorization was necessarily pragmatic rather than absolute. Finally, this study did not evaluate how patients interpret, emotionally respond to, or act upon AI-generated information, an important limitation given the psychosocial vulnerability often associated with sexual dysfunction and chronic pelvic pain syndromes. These factors underscore the need for cautious interpretation of AI performance metrics in rare disease contexts.

Future research should build on these findings by expanding evaluations to include multiple LLMs and repeated assessments over longer time intervals to better characterize temporal variability and model evolution. Incorporating retrieval-augmented architectures grounded in peer-reviewed medical databases may help reduce hallucinations and improve transparency of evidence sourcing, particularly for rare conditions with limited primary data. Further studies should also examine patient-centered outcomes, including comprehension, perceived credibility, anxiety modulation, and downstream health behaviors following exposure to AI-generated information. In addition, the development of standardized evaluation frameworks tailored to rare diseases accounting explicitly for evidence scarcity and clinical uncertainty would improve comparability across studies and reduce subjectivity in expert grading. Ultimately, interdisciplinary collaboration between clinicians, AI developers, and ethicists will be essential to define appropriate use cases for AI-driven health information tools and ensure that such technologies augment rather than undermine evidence-based patient care.

### Conclusions

In this exploratory evaluation, ChatGPT demonstrated stable readability and high short-term reproducibility when addressing patient-generated questions regarding HFS, although response accuracy varied according to clinical domain and occasionally extended beyond the available evidence base. These findings highlight both the promise and the current limitations of LLMs as accessible sources of health information for rare diseases. As AI becomes increasingly integrated into patient information seeking, robust validation frameworks, expert oversight, and continued refinement of medically grounded models will be essential to ensure that these technologies complement rather than replace evidence-based clinical care.

## Supplementary material

10.2196/93849 Multimedia Appendix 1 AI-generated responses T1.

10.2196/93849 Multimedia Appendix 2 AI-generated responses T2.

10.2196/93849 Multimedia Appendix 3 Reviewer ratings.

## Abbreviations

GFI: Gunning Fog Index
HFS: hard flaccid syndrome
ICC: intraclass correlation coefficient
IRR: interrater reliability
LLM: large language model
