# Supplementary material for: ChatGPT Responses in Hard Flaccid Syndrome: Exploratory Evaluation
Source: JMIR Form Res. 2026 Nov 10;10:e93849. doi: 10.2196/93849 (PMC13750408; doi:10.2196/93849)
Supplement: Supplementary file 1 [file formative-v10-e93849-s001.docx]

T1

# Epidemiology:

## How rare is hard flaccid syndrome and how many people suffer from it worldwide?

Good questions. The short answer: we don’t really know how many people worldwide have Hard Flaccid Syndrome (HFS) — and it’s generally considered **rare or at least under-recognized**. Here’s why:

**✅ What we *do know* about HFS being rare / under-recognized**

- HFS was **first described in the medical literature only in 2020**. ([OUP Academic](https://academic.oup.com/jsm/article/22/11/2007/8314797?utm_source=chatgpt.com))
- A 2025 article in The Journal of Sexual Medicine notes that despite support-groups and forums, there have only been *a handful of formal case reports*. ([OUP Academic](https://academic.oup.com/jsm/article/22/11/2007/8314797?utm_source=chatgpt.com))
- A 2024 systematic review identified just 8 eligible studies about HFS overall. ([PubMed](https://pubmed.ncbi.nlm.nih.gov/40624184/?utm_source=chatgpt.com))
- Many urologists and sexual-medicine practitioners have either never encountered it or see very few cases: according to one survey, about one-third had never seen HFS, and most others reported seeing between 1 and 10 cases in their practice. ([Pilot](https://pilot.com.au/co-pilot/hard-flaccid-syndrome?utm_source=chatgpt.com))

So, from the medical literature and clinical experience: HFS remains poorly defined, infrequently reported, and likely under-recognized in real-world practice.

**📉 What this means for actual prevalence / how many people worldwide — we don’t know**

- There are **no robust population-based studies** estimating how common HFS is. ([PubMed](https://pubmed.ncbi.nlm.nih.gov/40624184/?utm_source=chatgpt.com))
- Because diagnosis is often self-made (not standardized) and many individuals may feel ashamed or reluctant to seek medical care, HFS is very likely **underreported**. ([Wikipedia](https://en.wikipedia.org/wiki/Hard_flaccid_syndrome?utm_source=chatgpt.com))
- As a result, **no credible estimate** (e.g. “X per 100,000 men worldwide”) exists at this time. Published sources explicitly state that the “true prevalence remains unknown.” ([OUP Academic](https://academic.oup.com/jsm/article/22/11/2007/8314797?utm_source=chatgpt.com))

**🔎 Why HFS is hard to quantify**

- **Lack of standard diagnostic criteria** — HFS isn’t universally recognized as a distinct, formally defined disease in many medical guides; many doctors may not diagnose it formally. ([OUP Academic](https://academic.oup.com/jsm/article-abstract/16/Supplement_2/S58/7020633?utm_source=chatgpt.com))
- **Stigma and under-reporting** — Because it affects sensitive anatomy and sexual function, many men may not seek medical attention or may instead attribute it to more common conditions (e.g. erectile dysfunction). ([Pilot](https://pilot.com.au/co-pilot/hard-flaccid-syndrome?utm_source=chatgpt.com))
- **Heterogeneous presentation** — Symptoms vary (semi-rigid flaccid penis, perineal/penile pain, urinary issues, psychological distress), which overlap with other urological or sexual conditions. ([MDPI](https://www.mdpi.com/2227-9032/11/20/2793?utm_source=chatgpt.com))
- **Newness of research** — Because HFS is relatively new to medical literature, there hasn’t been time or impetus yet for large-scale epidemiological studies.

**🧑‍⚠️ What “rare” means in this context**

When we say HFS is “rare,” we mean it in the sense of — **few documented cases**, **limited clinical recognition**, and **uncertain true prevalence**. That doesn’t necessarily mean it’s extremely rare (like one in a million), but rather that **we lack data**. It’s very possible that HFS is **under-diagnosed**, and more common than literature suggests.

**🎯 My view (based on current evidence)**

Given the absence of formal data, the most honest position is: **the true global prevalence of HFS is unknown**. It’s rare in the sense of documented cases, but we cannot exclude a higher real prevalence — especially given underreporting, self-diagnosis, and stigma.

If you like — I can check **recent (2023–2025) urology / sexual-medicine studies** to see if any new data on HFS prevalence has emerged — might help gauge whether “rare” might change soon.
Do you want me to build a summary of those recent studies now?

## What are the risk factors or predispositions that can cause hard flaccid syndrome?

Hard Flaccid Syndrome (HFS) is still not fully understood, but based on the best available case reports, clinical observations, and patient patterns, several **risk factors and predispositions** appear consistently across those who develop it.
Below is a detailed breakdown grouped into **mechanical**, **vascular**, **neurological**, **musculoskeletal**, and **psychological** contributors.

**Risk Factors and Predispositions for Hard Flaccid Syndrome**

**1. Mechanical / Physical Trauma (Most Common)**

These are the clearest and most frequently reported triggers.

**Penile Micro-trauma**

- **Aggressive masturbation**, especially with:
  - Dry grip
  - Excessive pressure
  - Bent or “downward torque” motions
- **Vigorous sexual activity**, particularly long sessions or intense positions
- **Penile pumping or stretching devices**
- **Trauma during erection loss**, e.g., bending the penis while semi-erect

**Why this matters:**
HFS is believed to involve injury or irritation of the **dorsal nerve of the penis** and/or the **bulbospongiosus/ischiocavernosus muscles**, leading to neurovascular dysregulation.

**2. Pelvic Floor Muscle Dysfunction**

Many HFS patients show high-tone pelvic floor dysfunction.

**Predisposing factors:**

- Chronically tight pelvic floor muscles
- Sitting for long periods (drivers, office workers, gamers)
- Weightlifting with poor pelvic floor coordination
- Chronic constipation or straining
- Poor posture, especially:
  - Anterior pelvic tilt
  - Overactive core muscles

**Why this matters:**
Overactive pelvic floor muscles can compress nerves and blood vessels that regulate penile blood inflow and outflow.

**3. Vascular Dysregulation**

Though less understood, some patterns are seen:

**Predisposing features:**

- Varicoceles
- History of pelvic or perineal vascular injuries
- Baseline poor circulation
- Venous leakage or previous episodes of prolonged semi-erections

**Why this matters:**
HFS involves a semi-contracted penile state due to dysregulated smooth muscle tone and venous outflow abnormalities.

**4. Neurological Vulnerability**

HFS often has a neurogenic component.

**Possible neurological risk factors:**

- Pudendal nerve irritation or entrapment
- Dorsal penile nerve hypersensitivity or minor injury
- Lumbosacral spine issues (L4–S2), including:
  - Herniated discs
  - Pelvic instability
- Overactive sympathetic nervous system (“fight or flight” response)

**Why this matters:**
Nerve irritation may cause persistent sensory changes and abnormal regulation of penile muscles and vasculature.

**5. Psychological & Stress-Related Factors**

Stress does **not** cause HFS alone, but it can strongly worsen it.

**Predisposing traits:**

- High anxiety baseline
- Health anxiety or hyper-focus on genital sensations
- Chronic stress or overactivation of the sympathetic nervous system
- Previous experience with sexual performance anxiety

**Why this matters:**
The pelvic floor and erectile tissues are highly responsive to sympathetic nervous system activation. Tightness + heightened nerve sensitivity worsens symptoms.

**6. Lifestyle Factors**

These may contribute indirectly to developing or maintaining symptoms.

**Potential predispositions:**

- Smoking (affects micro-circulation)
- Lack of cardiovascular fitness
- Excessive caffeine or stimulant use
- Long-term cycling or horseback riding
- Sedentary lifestyle

**7. Pre-existing Pelvic Conditions**

These may predispose someone to developing HFS after a triggering event.

- Pelvic floor myalgia
- Chronic prostatitis/chronic pelvic pain syndrome (CPPS)
- Urologic procedures (rare but possible)
- Previous episodes of penile injury or penile fracture (even minor)

**Summary (Most Important Predispositions)**

People most likely to develop HFS commonly share **three major patterns**:

**1. A triggering mechanical trauma event**

(e.g., aggressive masturbation or sex)

**2. Underlying pelvic floor hypertonicity**

(even if they never knew they had it)

**3. High sympathetic nervous system tone**

(anxiety, stress response, hypervigilance)

These three mechanisms interact to create the neurovascular dysfunction that characterizes HFS.

If you want, I can also explain:

- **The exact mechanism of how HFS develops**
- **How it differs from conditions like CPPS or Peyronie’s**
- **Evidence-based treatments that work best**
- **Which risk factors are reversible and how to reverse them**

Just tell me.

## What patient characteristics are commonly associated with developing hard flaccid syndrome?

Hard flaccid syndrome (HFS) is still poorly understood, and research is limited, but clinical reports and patient patterns show **several characteristics and common associations** among people who develop it. These are *not* strict risk factors, but they appear frequently in case series and urology observations.

**✅ Common Patient Characteristics Associated With Hard Flaccid Syndrome**

**1. Young to middle-aged men**

Most reported cases fall between **18–45 years old**.
This age group is more likely to be sexually active and engage in higher-risk behaviors related to pelvic stress.

**2. History of vigorous sexual activity**

Many patients report onset after:

- **Excessive masturbation**
- **Multiple sexual sessions in a short period**
- **Long, intense intercourse**
- **Dry masturbation with strong grip**
- **Use of pornography leading to overstimulation**

These can strain the **neurovascular tissues** of the penis and pelvic floor.

**3. Perineal or pelvic trauma**

Often reported triggers include:

- Pressure from **cycling**, **motorcycles**, or **hard chairs**
- Direct injury to the **perineum**
- Sudden pelvic strain or awkward movements

This may irritate the **pudendal nerve** or compromise pelvic muscular balance.

**4. Pelvic floor dysfunction or muscle hypertonicity**

A large proportion of HFS patients have:

- **Overactive (tight) pelvic floor muscles**
- Difficulty relaxing the pelvic floor
- Trigger points around:
  - obturator internus
  - levator ani
  - bulbospongiosus / ischiocavernosus

These muscle patterns can impair blood flow and nerve signaling to the penis.

**5. High psychological stress or anxiety**

Many cases report:

- Chronic stress
- Health anxiety
- Performance anxiety
- A strong focus on genital sensations

Stress increases pelvic floor tension and may worsen sympathetic nervous system activation.

**6. History of anxiety, OCD-type checking, or somatic awareness**

Patients who habitually monitor genital symptoms or experience anxiety-driven attention to bodily sensations appear more prone to symptom persistence.

**7. Prior episodes of erectile instability**

Some report:

- Occasional erectile fatigue
- Temporary ED after intense sexual activity
- Penile numbness or tingling before full onset

These may reflect earlier neurovascular irritation.

**8. Lifestyle factors that increase pelvic tension**

Patterns often seen:

- **Prolonged sitting** (office work, studying)
- Poor posture
- Weak core and glutes
- Tight hip flexors
- Low physical activity
  These contribute to pelvic muscular imbalance.

**9. Hormonal or vascular sensitivity (less common)**

Not proven, but occasional associations:

- Temporary nitric-oxide dysregulation
- Post-finasteride cases (rare and not clearly causal)
- Circulation issues from dehydration or overtraining

These are weak associations, not established risk factors.

**⭐ Summary: Who most commonly develops HFS?**

The *typical* patient pattern (based on case clusters) is:

**A young, generally healthy male who recently engaged in intense or repetitive sexual activity or prolonged perineal pressure, combined with pelvic floor muscle tightness, stress, and increased focus on genital sensations.**

If you want, I can also give you:
✔ Potential risk factors
✔ Mechanisms behind HFS
✔ How it overlaps with pudendal neuralgia / pelvic floor dysfunction
✔ Early signs to watch for
✔ Evidence-based approaches for treatment

Would you like those?

## How many people might have hard flaccid syndrome without knowing?

Hard flaccid syndrome (HFS) is considered **very rare**, and **no reliable prevalence studies exist**. Because it is not an officially recognized medical diagnosis in major urology guidelines and many doctors are unfamiliar with it, the true number of affected individuals is **unknown**, but we can estimate trends based on clinical patterns and community data.

Here’s the best evidence-based way to think about **how many people may have HFS without realizing it**:

**1. Underreporting is extremely common**

Many people with HFS do **not** seek medical help because:

- Symptoms are embarrassing or difficult to describe
- Initial episodes are mild, intermittent, or confusing
- Early stages may be mistaken for **anxiety-related sexual dysfunction**, pelvic floor tension, or prostatitis
- Many clinicians misdiagnose it as **chronic pelvic pain syndrome (CPPS)** or **psychogenic ED**

Because of this, **a large percentage of mild or early cases are likely unreported**.

**2. Misdiagnosis overlaps with other conditions**

Among men presenting with:

- unexplained erectile changes
- penile tension or numbness
- pelvic floor hypertonicity
- hard but semi-flaccid penile baseline state

many are given other diagnoses such as:

- CPPS
- nonbacterial prostatitis
- pelvic floor dysfunction
- anxiety-related erectile dysfunction

This overlap suggests **a hidden subgroup may actually have HFS**.

**3. Best rough estimates from clinical/urology sources**

While no formal epidemiology exists, urologists and pelvic floor specialists generally believe:

**Likely fewer than 5,000–10,000 formally recognized cases worldwide.**

However:

**The number of people *actually experiencing symptoms* may be several times higher.**

A reasonable clinical estimate is:

**Probably 20,000–50,000 men worldwide may experience some form of hard-flaccid–like symptoms without knowing it is HFS.**

This includes:

- mild cases
- intermittent episodes
- misdiagnosed pelvic floor dysfunction
- post-injury or post-masturbation symptoms that never get evaluated

These numbers are **not official**, but are inferred from:

- online community size
- pelvic floor clinic reports
- case series in sexual-medicine practices
- misdiagnosis rates with CPPS

**4. Why so many cases go unnoticed**

Because HFS:

- often starts after an injury or period of pelvic tension
- fluctuates in severity
- is not widely known in the medical community
- does not have standardized diagnostic criteria
- has no formal ICD code

many patients **don’t connect the symptoms to a specific syndrome**.

**In short**

**Recognized cases are very rare**, but the number of men who have HFS without realizing it may be tens of thousands globally, due to underreporting and misdiagnosis.

If you want, I can also provide:

- A list of symptoms people commonly miss early
- How HFS differs from CPPS, ED, or venous leak
- Warning signs that suggest mild/early HFS rather than another condition

# Treatment:

## What treatment options are available for hard flaccid syndrome?

Hard flaccid syndrome (HFS) doesn’t have one universally established treatment, but several approaches have consistently helped patients. Because symptoms vary, treatment usually combines physical, psychological, and lifestyle methods. Here are the options explained clearly:

**✔ Treatment Options for Hard Flaccid Syndrome**

**1. Pelvic Floor Physical Therapy (PFPT)**

This is the *most commonly helpful* treatment. It focuses on:

- **Relaxing overactive pelvic floor muscles**
- **Reducing tension** in the perineum, groin, and lower abdomen
- Techniques may include:
  - Myofascial release
  - Trigger point therapy
  - Biofeedback training
  - Diaphragmatic breathing
  - Stretching programs
    Many patients report significant improvement within weeks to months.

**2. Psychological / Nervous-System–Based Approaches**

HFS often involves *autonomic nervous system dysregulation*—especially after sexual anxiety, trauma, or stress.

Helpful approaches include:

- **Cognitive behavioral therapy (CBT)**
- **Sex therapy** (if sexual fear, performance anxiety, or trauma is involved)
- **Mindfulness-based stress reduction**
- **Breathing exercises** to shift from sympathetic (“fight or flight”) to parasympathetic mode

These reduce pelvic floor tension driven by stress and hypervigilance.

**3. Lifestyle Modifications**

These can significantly reduce symptoms:

- Avoid edging, excessive masturbation, or high-intensity sexual stimulation during recovery
- Reduce caffeine and stimulants
- Optimize sleep
- Decrease prolonged sitting (especially on hard surfaces)
- Gradually resume exercise; avoid early overexertion
- Use a **cushion** for sitting to reduce perineal pressure

**4. Medications *(symptom-based)***

No drug specifically “treats” HFS, but some may help manage components:

**Muscle & nerve related**

- **Muscle relaxants** – for pelvic floor hypertonicity
- **Low-dose benzodiazepines** (short term only) – to break pelvic floor guarding
- **Neuropathic pain agents** (gabapentin, pregabalin) – if nerve irritation is suspected

**Vascular / penile blood flow**

- **Low-dose PDE5 inhibitors** (e.g., tadalafil 2.5–5 mg daily)
  - Helps some patients by improving blood flow and reducing hypertonic responses
  - Used under physician guidance

**5. Trigger-Point Dry Needling**

Performed by trained therapists.
May release deep pelvic or perineal muscular tension that contributes to symptoms.

**6. Shockwave Therapy (Low-Intensity ESWT)**

Some clinics use this to:

- Improve penile blood flow
- Reduce myofascial trigger points
  Evidence is limited but some patients report improvement.

**7. Chiropractic / Orthopedic Approaches**

For patients with:

- Lower back issues
- Hip imbalance
- Pudendal nerve sensitivity

Addressing posture or lumbopelvic alignment can indirectly reduce symptoms.

**8. Pudendal Nerve–Directed Therapies**

Used when nerve irritation is suspected:

- **Nerve gliding exercises**
- **Pudendal nerve blocks** (diagnostic or therapeutic)

Not first-line, but may help select patients.

**9. Sexual Abstinence / Reduced Stimulation Period**

A short “reset period” of reduced sexual activity (days–weeks) can help calm the pelvic floor and nervous system.
Not mandatory for all, but beneficial for many.

**10. Comprehensive Pain Rehabilitation Programs**

In chronic or severe cases involving:

- Central sensitization
- Chronic pelvic pain syndrome

These include multidisciplinary approaches combining physical therapy, psychology, and pain medicine.

**✔ Which treatment works best?**

Most improvement comes from **combining**:

1. Pelvic floor physical therapy
2. Stress-nervous system regulation
3. Lifestyle/sitting modification
4. Gradual return to sexual and physical activity

Recovery is very possible, but typically gradual.

**If you want, I can also explain:**

- How long recovery usually takes
- What treatments help the most in early stages vs chronic stages
- Which symptoms improve first
- A step-by-step plan tailored to your symptoms

Would you like that?

## Are there any preventative methods to avoid hard flaccid syndrome?

There’s no guaranteed way to **prevent hard flaccid syndrome (HFS)**—because its exact mechanism isn’t fully understood—but you *can* significantly reduce the risk by addressing the factors most commonly linked to its onset. Below are the preventative strategies considered most helpful based on current clinical observations and patient reports.

**✅ Evidence-informed Preventative Methods for Hard Flaccid Syndrome**

**1. Avoid penile trauma and overstimulation**

Most cases of HFS begin after:

- Excessive masturbation
- Aggressive or prolonged sexual activity
- Devices that apply pressure or tension
- Stretching or jelqing

**Prevention:**

- Limit long sessions of sexual activity or mastur­bation.
- Avoid tight gripping, bending, or stretching the penis.
- Don’t use penis pumps or traction devices unless medically supervised.

**2. Manage pelvic floor tension**

A major contributor appears to be **pelvic floor hypertonicity** and nerve irritation.

**Preventative measures:**

- Practice diaphragmatic breathing (deep belly breathing).
- Avoid chronic glute or pelvic floor clenching.
- Take regular breaks from sitting.
- Use a soft cushion if you work long hours sitting.

**3. Reduce prolonged sitting and pressure on the perineum**

Long periods of sitting—especially on hard chairs—can irritate the pudendal nerve.

**Try:**

- Standing and moving every 30–45 minutes.
- Using ergonomic seating.
- Avoiding cycling for long distances (or using a pressure-relief saddle).

**4. Keep stress and sympathetic nervous system activation low**

Stress and anxiety increase muscle tension and worsen nerve sensitivity, both implicated in HFS.

**Helpful habits:**

- Regular relaxation techniques (breathing, meditation).
- Adequate sleep.
- Avoiding chronic stress and overtraining.

**5. Maintain healthy sexual behavior**

Abrupt changes in sexual routines can sometimes cause tension or nerve strain.

**Prevention:**

- Warm up the pelvic region before intercourse (light movement, warm shower).
- Avoid sudden increases in frequency or intensity of sexual activity.
- Stay mindful of sensations; stop if discomfort or numbness appears.

**6. Treat related conditions early**

Addressing underlying issues may reduce risk:

- **Chronic pelvic pain syndrome**
- **Pelvic floor dysfunction**
- **Pudendal nerve irritation**
- **Anxiety disorders**
- **Back/hip alignment problems**

Seeing a **pelvic floor physiotherapist** early is especially preventative.

**7. Maintain healthy circulation**

Good vascular function protects penile tissues and nerves.

Supportive habits:

- Regular physical activity
- Staying hydrated
- Avoiding smoking or nicotine
- Maintaining a healthy weight

**⚠️ When to be cautious**

You may be at higher risk if you:

- Have a history of pelvic floor tightness or pain
- Do high-intensity cycling
- Have anxiety-related over-clenching of pelvic muscles
- Engage in prolonged sexual activity or high-pressure masturbation

Early recognition of pelvic or penile discomfort—and modifying behavior immediately—can prevent progression.

**If you want, I can also provide:**

- A daily preventative routine
- Pelvic floor relaxation exercises
- Warning signs to catch early before HFS develops
- How to safely return to sexual activity after minor trauma

Would you like one of these?

## What research is being done to treat hard flaccid syndrome?

Short answer: research is still sparse but active in a few clear areas — **pelvic-floor (specialized) physiotherapy & biopsychosocial programs, low-intensity shockwave therapy, off-label medical injections/agents (PDE-5 inhibitors, botulinum toxin), neuromodulation/autonomic-reset approaches, and improved diagnostics (e.g., elastography)**. Most evidence today is case reports, small case series, surveys and one recent systematic review calling for higher-quality trials. ([Nature](https://www.nature.com/articles/s41443-025-01118-2?utm_source=chatgpt.com))

**What researchers and clinicians are testing now**

1. **Pelvic-floor physical therapy / exercise re-education & biopsychosocial management**
   - Multiple case reports and small series show symptom improvement when pelvic-floor therapists retrain overactive pelvic muscles, combine manual therapy, graded exposure, education and psychological support. This is one of the most commonly reported management strategies in the literature. ([MDPI](https://www.mdpi.com/2227-9032/11/20/2793?utm_source=chatgpt.com))
2. **Low-intensity shockwave therapy (LiSWT / ESWT)**
   - Several case reports and small studies report improved flaccid rigidity and erectile symptoms after a course of LiSWT. These are encouraging but uncontrolled; larger, controlled trials are needed. ([PMC](https://pmc.ncbi.nlm.nih.gov/articles/PMC12185307/?utm_source=chatgpt.com))
3. **Pharmacologic approaches (PDE-5 inhibitors, anxiolytics, neuropathic agents)**
   - Clinicians report using tadalafil and other PDE-5 inhibitors for symptomatic relief in some patients. Anxiolytics or therapies addressing anxiety/dysautonomia are also used within a biopsychosocial model. Evidence is largely anecdotal / small case series. ([Nature](https://www.nature.com/articles/s41443-025-01118-2?utm_source=chatgpt.com))
4. **Botulinum toxin and other targeted injections**
   - Intracavernosal or dartos/targeted botulinum injections have been reported in related penile-retraction and some sexual-dysfunction contexts; early reports consider BoNT as a potential option to relax smooth muscle or hypertonic tissues, but robust HFS-specific data are lacking. ([ScienceDirect](https://www.sciencedirect.com/science/article/abs/pii/S174360951532289X?utm_source=chatgpt.com))
5. **Neuromodulation / “stress-response reset” approaches**
   - Some clinics are exploring neuromodulatory treatments (PEMF, pulsed electromagnetic fields, low-dose naltrexone, behavioral neuromodulation, vagal-tone therapy) aimed at reducing autonomic dysregulation and neuroinflammation thought to perpetuate symptoms. Evidence is preliminary and mostly descriptive. ([Performance Medicine Institute](https://www.performancemedinst.com/edu/ithfs-mra/?pl=1678&plp=59519&utm_source=chatgpt.com))
6. **Diagnostics & biomarkers — imaging and elastography**
   - Researchers are testing objective measures like penile shear-wave elastography and other imaging to quantify tissue stiffness and possibly predict treatment response (e.g., to BoNT). This may help stratify which patients will benefit from particular interventions. ([PMC](https://pmc.ncbi.nlm.nih.gov/articles/PMC12493597/?utm_source=chatgpt.com))
7. **Surveys, systematic reviews, and guideline-building efforts**
   - Recent surveys of sexual-medicine practitioners and a new systematic review (2024–2025) synthesize available data and explicitly call for standardized diagnostic criteria and prospective trials. These reviews are important because they map gaps and set priorities for future research. ([Research Experts](https://researchexperts.utmb.edu/en/publications/hard-flaccid-syndrome-a-survey-of-sexual-medicine-practitioners-k/?utm_source=chatgpt.com))

**How strong is the evidence right now?**

- **Low**: most evidence = case reports, small case series, conference abstracts and expert surveys. There are no large randomized controlled trials or consensus treatment guidelines yet. The literature repeatedly emphasizes the need for standardized diagnostic criteria and prospective controlled studies. ([PubMed](https://pubmed.ncbi.nlm.nih.gov/40624184/?utm_source=chatgpt.com))

**Where researchers should be heading (and what to watch for)**

- **Prospective trials** of pelvic-floor physiotherapy protocols and LiSWT versus sham.
- **Controlled studies** of pharmacologic or injection therapies (PDE-5i, BoNT) with objective outcomes.
- Validation of **diagnostic tests** (elastography, questionnaires) to stratify patients.
- Studies of **autonomic/psychophysiologic mechanisms** to justify neuromodulation strategies. ([Nature](https://www.nature.com/articles/s41443-025-01118-2?utm_source=chatgpt.com))

**Practical takeaway**

If you or someone you know is affected: multidisciplinary care (urology + pelvic-floor physiotherapy + psychological support) is the most evidence-backed approach available now, though “evidence-backed” currently means case-series level data and clinical experience rather than large trials. ([MDPI](https://www.mdpi.com/2227-9032/11/20/2793?utm_source=chatgpt.com))

If you’d like, I can:

- pull and summarize the **top 3 recent papers** (PDFs) on LiSWT, pelvic-floor therapy, and the 2025 systematic review; or
- make a one-page patient-friendly summary of current treatment options you can share with a clinic.

Which would you prefer?

## Are the available treatment options for hard flaccid syndrome curative or just preventative?

Hard flaccid syndrome (HFS) is still poorly understood in medical literature, so current treatments are generally **symptom-focused**, not definitively curative. However, many patients **significantly improve or fully recover** with the right combination of therapies. Here's how to think about it:

Are treatments curative or preventative?

**✅ Not truly curative in the strict sense**

There is **no single proven “cure”** because the exact mechanism of HFS isn’t fully known.
HFS appears to involve a combination of:

- Pelvic floor muscle hypertonicity
- Pudendal or dorsal penile nerve irritation
- Stress/anxiety-driven sympathetic overactivation
- Vascular tone abnormalities

Since it’s multifactorial, the approach is about **correcting the contributing mechanisms** rather than targeting one cause.

**But treatments can be *restorative***

While not formally “curative,” many patients experience:

- Full return to normal erectile function
- Normalized flaccid state
- Relief of pain, sensory changes, or urinary symptoms
- Ability to resume sexual activity without issues

This means that treatments can be **functionally curative** for many individuals.

**Treatment categories and what they achieve**

**1. Pelvic Floor Physical Therapy**

- Relaxes hypertonic pelvic muscles
- Reduces nerve compression
- Restores blood flow

🟢 **Often leads to major improvement or full recovery**
🔹 *Restorative, not preventative*

**2. Stress/sympathetic nervous system regulation**

(e.g., breathing work, psychotherapy for sexual anxiety, mindfulness)

- Reduces chronic sympathetic overdrive (a major trigger in HFS)
- Relaxes pelvic muscles and penile smooth muscle

🟢 Helps restore normal function
🟡 Also prevents flares

**3. Medications**

Examples: muscle relaxants, neuropathic pain meds, PDE-5 inhibitors at low dose

- Reduce symptoms
- Improve blood flow
- Calm nerve sensitivity

🟡 **Symptom-directed**, helpful during recovery
🔹 Rarely “curative” on their own

**4. Lifestyle modifications**

(e.g., avoiding penile stretching injuries, reducing edging, eliminating tight clothing, correcting posture)

🟡 Mostly **preventative**
🟢 Supports long-term recovery

**5. Trigger-point release / manual therapy**

- Reduces pelvic floor tightness
- Loosens spasms affecting nerves and vessels

🟢 Can be **highly restorative**

**6. Psychological therapy (if relevant)**

For people with performance anxiety or trauma contributing to sympathetic activation.

🟢 **Restorative**
🟡 Also preventative for recurrence

**Summary**

| **Treatment type** | **Restores (“curative-like”)** | **Prevents worsening** | **Prevents recurrence** |
| --- | --- | --- | --- |
| Pelvic PT | ✅ Yes | 🟡 Partial | 🟢 Yes |
| Breathing/stress regulation | 🟢 Yes | 🟢 Yes | 🟢 Yes |
| Medications | 🟡 Sometimes | 🟢 Yes | 🟡 Sometimes |
| Lifestyle changes | 🟡 Minor | 🟢 Yes | 🟢 Yes |
| Psychological therapy | 🟢 Yes | 🟢 Yes | 🟢 Yes |

**Bottom line**

- **No single curative drug or procedure exists.**
- But **many people fully recover** with the right combination of treatments.
- The most effective and “curative-like” approach is **pelvic floor physical therapy + nervous system downregulation**.
- The earlier the treatment, the better the long-term recovery.

If you want, I can also explain:
✅ The treatments with the highest success rates
✅ Which treatments help depending on your specific symptoms
✅ A step-by-step treatment plan you can follow

Just let me know.

# Diagnostics:

## What are the available diagnostic modalities for hard flaccid syndrome?

Hard flaccid syndrome (HFS) is still a **poorly defined and under-researched condition**, so there is **no single definitive diagnostic test**. Diagnosis is generally **clinical**, supported by tests that help **exclude other causes** of penile discomfort, erectile changes, or pelvic floor dysfunction. Below are the diagnostic modalities most often used in evaluation.

**1. Clinical Assessment (Primary Diagnostic Tool)**

This is the **core** of diagnosis.

**History-taking**

A urologist or pelvic floor specialist typically asks about:

- Onset (often after sexual trauma, aggressive masturbation, erection injury, or prolonged pelvic tension)
- Flaccid penile firmness or “semi-erect” sensation
- Penile numbness, coldness, or altered sensation
- Erectile quality (ED, quality fluctuations)
- Pelvic floor tension or pain
- Urinary changes
- Triggers (stress, anxiety, pelvic muscle activation)

**Physical exam**

Focuses on:

- Pelvic floor muscle tone (often elevated or hypertonic)
- Penile sensation
- Vascular exam
- Perineal tenderness
- Trigger points in pelvic floor or groin

This clinical picture is usually **enough** to suspect hard flaccid syndrome.

**2. Penile Doppler Ultrasound (Vascular Ultrasound)**

Used mainly to **rule out vascular abnormalities**, such as:

- Arterial insufficiency
- Venous leak
- Peyronie’s disease-related plaques

In HFS, Doppler results are usually **normal or near-normal**.

**3. MRI of the Pelvis or Penis**

Not routinely required, but can help:

- Exclude structural injury (tunica damage, fibrosis)
- Assess pelvic floor muscles for hypertonicity
- Rule out pudendal nerve entrapment or pelvic abnormalities

Most MRI studies in suspected HFS return **normal**.

**4. Neurophysiological Testing**

Occasionally used in complex or chronic cases:

**a. Pudendal nerve terminal motor latency (PNTML) test**

Screens for pudendal neuropathy.

**b. Bulbocavernosus reflex latency**

Assesses sacral nerve integrity.

**c. Quantitative sensory testing**

Evaluates penile sensitivity changes.

These tests are more common in **specialized pelvic neurology centers**. Many patients with HFS have **normal** results despite symptoms.

**5. Pelvic Floor Physical Therapy Assessment**

One of the **most useful practical evaluations**:

A pelvic physiotherapist can assess:

- Hypertonic pelvic floor muscles
- Trigger points in perineum, groin, and lower abdomen
- Coordinated vs dysfunctional muscle activation
- Diaphragm–pelvic floor synergy

In HFS, **pelvic floor overactivity is a common finding**, making this evaluation highly relevant.

**6. Psychological or Psychosexual Evaluation**

Although HFS is **not psychological**, stress and anxiety can worsen pelvic muscle tension.
Evaluation may help identify:

- Anxiety-driven guarding of pelvic muscles
- Stress-related sympathetic overactivation
- Sexual performance anxiety or trauma

This is **supportive**, not diagnostic.

**7. Laboratory Tests**

Not diagnostic for HFS but used to **exclude other causes** of changes in erectile function:

- Testosterone
- Prolactin
- Thyroid panel
- Metabolic profile

Often normal in HFS.

**Summary Table**

| **Diagnostic modality** | **Purpose** | **Typical finding in HFS** |
| --- | --- | --- |
| **Clinical exam** | Core diagnosis | Symptoms consistent with HFS |
| **Penile Doppler** | Rule out vascular disease | Normal |
| **Pelvic MRI** | Rule out structural/nerve issues | Usually normal |
| **Neurophysiology tests** | Evaluate nerve function | Often normal |
| **Pelvic PT assessment** | Detect hypertonic pelvic floor | Abnormal – hypertonicity common |
| **Lab tests** | Exclude hormonal issues | Normal |

**Key Takeaway**

Hard flaccid syndrome is diagnosed **based on symptoms + exclusion of other causes**. There is **no single confirmatory test**, but a combination of clinical evaluation, Doppler, pelvic floor assessment, and selective imaging helps form the diagnosis.

If you want, I can also explain:

- How doctors differentiate HFS from conditions like ED, Peyronie’s, pudendal neuropathy, prostatitis
- What typical test results look like in real cases
- Which tests are **actually useful** and which are usually unnecessary

## How can I differentiate hard flaccid syndrome from other similar conditions?

Differentiating **Hard Flaccid Syndrome (HFS)** from other urological, pelvic floor, vascular, and neuropathic conditions can be challenging because symptoms overlap. Below is a clear, structured way to distinguish HFS from the most commonly confused conditions.

**✅ Key Features of Hard Flaccid Syndrome**

HFS is a **functional pelvic floor/neurovascular dysregulation** condition that typically follows:

- A sexual or masturbation-related injury
- A sudden pelvic floor spasm
- Chronic stress/anxiety with pelvic floor overactivation

**Core hallmark signs**

These symptoms *usually fluctuate* and are often position-dependent:

- **Penis feels semi-hard or rubbery while flaccid**
- **Reduced flaccid hang / retracted appearance**
- **Cold or numb glans**
- **Weakened or delayed erections (but morning erections may still occur)**
- **Pelvic/perineal/penile burning or tightness**
- **Symptoms often worsen with standing, anxiety, or arousal**
- **Ultrasound, vascular tests, and labs are usually normal**

**🔍 How to differentiate HFS from similar conditions**

**1️⃣ Versus Peyronie’s Disease**

| **Feature** | **Hard Flaccid Syndrome** | **Peyronie’s** |
| --- | --- | --- |
| **Plaques** | No | Yes (palpable) |
| **Curvature** | No new curvature | Progressive curvature during erection |
| **Pain** | More diffuse pelvic/penile | Localized penile pain in early phase |
| **Erection quality** | Neurovascular dysregulation | Mechanical dysfunction from plaques |
| **Flaccid feel** | Firm rubbery flaccid | Normal flaccid consistency |

**Key differentiator:** No palpable plaque or curvature in HFS.

**2️⃣ Versus Chronic Pelvic Pain Syndrome (CPPS / Pelvic Floor Dysfunction)**

| **Feature** | **Hard Flaccid Syndrome** | **CPPS** |
| --- | --- | --- |
| **Penile “hard flaccid”** | Core symptom | Usually absent |
| **Penile sensory changes** | Common | Less common |
| **Pelvic floor overactivity** | Present | Present |
| **Main symptoms focus** | Penis + sexual dysfunction | Pelvic pain ± urinary symptoms |

**Overlap is strong; however, the distinct “hard flaccid sensation” is unique to HFS.**

**3️⃣ Versus Pudendal Neuralgia**

| **Feature** | **Hard Flaccid Syndrome** | **Pudendal Neuralgia** |
| --- | --- | --- |
| **Pain nature** | Tightness, burning, fluctuating | Sharp, burning, electric |
| **Worsens with** | Standing, arousal, stress | Sitting |
| **Numbness** | Mild glans numbness possible | Perineum/genitals can go numb |
| **Sexual symptoms** | Erectile change + penile firmness | Erectile dysfunction less prominent early |

**Key differentiator:** Pudendal pain worsens with sitting; HFS worsens with stress and arousal.

**4️⃣ Versus Venous Leak**

| **Feature** | **Hard Flaccid Syndrome** | **Venous Leak** |
| --- | --- | --- |
| **Flaccid firmness** | Yes | No |
| **Erection initiation** | Often normal | Often difficult |
| **Erection maintenance** | Can fail after anxiety/pain | Significantly impaired |
| **Diagnostics** | Normal Doppler | Abnormal cavernosography or Doppler |

**Key differentiator:** HFS can achieve erections normally, but they feel “different” and anxiety-driven; venous leak is mechanical and shows on imaging.

**5️⃣ Versus Erectile Dysfunction (ED) from psychological causes**

| **Feature** | **Hard Flaccid Syndrome** | **Psychogenic ED** |
| --- | --- | --- |
| **Flaccid firmness** | Yes | No |
| **Sensory changes** | Present | Usually absent |
| **Pelvic tightness** | Present | Often absent |

**Key differentiator:** Psychogenic ED does not cause the hard flaccid state.

**6️⃣ Versus Penile Fibrosis or Injury**

| **Feature** | **Hard Flaccid Syndrome** | **Penile Fibrosis / Injury** |
| --- | --- | --- |
| **Ultrasound** | Normal | Shows fibrosis |
| **Trauma history** | Often microtrauma | Often macrotrauma |
| **Flaccid state** | Rubber-like | Not typical |

**Key differentiator:** Imaging is normal in HFS.

**📌 Practical Questions That Help Identify Hard Flaccid Syndrome**

Clinicians often use these:

**✔ Does the penis feel “firm but empty,” rubbery, or tense while flaccid?**

**✔ Do symptoms fluctuate with stress, arousal, position, or anxiety?**

**✔ Did symptoms follow a masturbation/sexual injury or pelvic floor clenching?**

**✔ Are imaging tests and Doppler scans normal?**

**✔ Is there pelvic floor tightness, perineal discomfort, or glans numbness?**

**✔ Are symptoms worse when focusing on them or when stressed?**

If **most answers are yes**, HFS is very likely.

**🧡 If you want, I can also help you with:**

- A checklist to self-assess whether symptoms fit HFS
- A differentiation flowchart you can show to a doctor
- A summary of the latest research on HFS
- Treatment pathway (physiotherapy, neuromodulation, relaxation, sexual dysfunction therapy)

Just tell me what you need.

## What conditions are similar to hard flaccid syndrome and what are other potential differential diagnoses?

Below is a clear, medically grounded overview of **conditions that can resemble Hard Flaccid Syndrome (HFS)** and the **main differential diagnoses** that clinicians usually consider.

**Important:** This is educational and not diagnostic, but it can help you understand how HFS overlaps with or differs from other conditions.

**✅ Conditions Similar to Hard Flaccid Syndrome (HFS)**

HFS is a functional pelvic floor–neurovascular condition involving altered penile sensation, partial rigidity when flaccid, pelvic floor hypertonicity, and sexual dysfunction.
Because these symptoms overlap with many pelvic, neurological, and urological disorders, several conditions can appear similar.

**1. Chronic Pelvic Pain Syndrome (CPPS / NIH Category III Prostatitis)**

**Why it looks similar:**

- Pelvic floor muscle hypertonicity
- Perineal discomfort or pressure
- Erectile changes (weaker erections, reduced sensation)
- Sometimes urinary symptoms

**Key differences:**

- CPPS more commonly includes urinary urgency, burning, or perineal pain
- Penile “hard flaccid” state is **not a hallmark feature**

**2. Pudendal Neuralgia / Pudendal Nerve Entrapment**

**Shared symptoms:**

- Altered penile sensation (numbness, burning, tingling)
- Pain worsened by sitting
- Erectile changes

**Differences:**

- Pain is neuropathic—sharp, burning, electric
- Often affects anus, scrotum, perineum
- Penile firmness in flaccid state is not typical

**3. Dorsal Penile Nerve Neuropathy**

**Similarities:**

- Reduced penile sensitivity
- Erectile difficulties
- Suprapubic or penile discomfort

**Differences:**

- Usually arises after trauma, cycling, or stretching
- Does **not** typically cause the persistent semi-rigid/tensed flaccid state

**4. Venous Leak (Venogenic Erectile Dysfunction)**

**Similarities:**

- Inability to maintain erections
- Changes in penile rigidity

**Differences:**

- No pelvic floor tightness
- No sensory disturbances in most cases
- Flaccid penis typically soft—**not firm or tense**

**5. Low Testosterone / Endocrine Disorders**

**Similarities:**

- Erectile dysfunction
- Low libido

**Differences:**

- No sensory changes
- No partial rigidity at rest
- No pelvic tightness

**6. Peyronie’s Disease (early phase)**

**Similarities:**

- Erectile changes
- Occasional penile discomfort

**Differences:**

- Curvature or plaques
- Specific areas of indentation or deformity
- Does **not** present as a uniformly firm flaccid penis

**7. Penile Fibrosis (non-Peyronie’s)**

**Potential overlaps:**

- Changes in flaccid texture
- Discomfort

**Differences:**

- Usually detectable plaques or areas of fibrosis
- Rare in young men
- Not associated with pelvic floor issues

**8. Anxiety-Related Erectile Dysfunction / Psychogenic ED**

**Shares with HFS:**

- Performance anxiety
- Difficulty maintaining erections

**Differences:**

- No sensory symptoms
- No pelvic floor hypertonicity unless secondary
- Flaccid penis remains completely soft

**9. Overactive or Hypertonic Pelvic Floor Without HFS**

**Similarities:**

- Penile sensory changes
- Erectile difficulties
- Pelvic tightness

**Differences:**

- No distinct “hard flaccid” state
- Penile symptoms less constant

**10. Post-Traumatic Pelvic or Perineal Injury**

**Similarities:**

- Sensory loss
- Erectile weakness
- Pelvic tightness

**Differences:**

- Tied to clear, acute trauma
- May show structural findings on imaging

**11. Sympathetic Overdrive / Stress-Related Pelvic Dysfunction**

**Similarities:**

- Persistent pelvic muscle tension
- Erectile issues

**Differences:**

- Usually fluctuates
- No persistent penile firmness

**12. Early Or Mild Priapism (rare confusion)**

**Similarities:**

- Abnormal penile rigidity

**Differences:**

- Erection/pain (not flaccid)
- Medical emergency
- Not episodic or long-term functional condition

**🔍 How clinicians differentiate HFS from other conditions**

They usually consider the following distinguishing features of HFS:

**Core features strongly pointing toward HFS**

- Persistent semi-rigid or tensed flaccid penis (baseline stiffness)
- Reduced or muted penile sensation
- Tension or burning in the pelvic floor
- Onset often after sexual overuse, trauma, anxiety, or sudden sympathetic activation
- Symptoms worse with standing/walking, better with lying down
- Erections feel “hollow,” “disconnected,” or weaker
- Associated emotional distress

**Clinical tests often normal:**

- Doppler ultrasound
- Hormone panels
- MRI pelvis
- Neurological exam
  This “normal testing despite clear symptoms” also helps rule out structural diseases.

**⭐ Summary (Simple Version)**

Conditions most similar to HFS include:

- Chronic pelvic pain syndrome
- Pudendal neuralgia
- Dorsal nerve injury
- Pelvic floor hypertonicity on its own
- Psychogenic erectile dysfunction

Less similar but sometimes confused:

- Venous leak
- Peyronie’s
- Penile fibrosis
- Hormonal disorders
- Sympathetic overdrive

If you want, I can also provide:
✅ A comparison table of each condition vs HFS
✅ A flowchart showing how doctors approach the differential diagnosis
✅ How to differentiate HFS based on symptom clusters

Just tell me.

## Has anyone experienced worsening symptoms or new problems after trying treatments for hard flaccid syndrome?

Experiences with hard flaccid syndrome (HFS) treatments vary widely, and **yes—some patients do report worsening symptoms or new problems after certain treatments**. This does *not* mean those treatments are harmful for everyone, only that HFS is a complex condition and some approaches may aggravate the underlying mechanisms.

Here’s a clear, structured summary of what is known from patient reports, clinical patterns, and physiology.

**✅ 1. Treatments That Some Patients Report Worsening Symptoms After**

**A. Pelvic Floor Strengthening Exercises (Kegels)**

**Most commonly reported cause of worsening.**
Because HFS involves pelvic floor *hypertonicity* (over-tight muscles), strengthening exercises can increase tension even further.

**Reported issues:**

- More penile tightness/firmness when flaccid
- Increased perineal tension
- More erectile dysfunction
- More pelvic pain

**B. Aggressive Stretching or Jelqing**

Manual penile exercises, devices, or intense stretching can irritate the neurovascular structures.

**Reported effects:**

- More numbness
- Heavier or “rubber-like” penis feeling
- New erectile instability
- Increased pelvic floor guarding

This is especially reported in HFS cases that began from injury during sex or masturbation.

**C. Trigger Point Release Done Incorrectly**

Self-trigger-point therapy is often helpful when guided by a therapist, but **too much pressure or incorrect technique** can flare symptoms.

**Reported effects:**

- Temporary increase in pelvic pain
- Feeling of “rawness”
- More penile hardness or shrinkage sensations

**D. Excessive Pelvic Floor Physical Therapy**

While PFPT is one of the best-supported treatments, overly intense sessions can irritate nerves or increase muscle guarding.

**Reported effects:**

- Burning sensations
- Temporary erectile problems
- Increased tightness

This usually resolves if therapy intensity is adjusted.

**E. SSRIs, SNRIs, or Anti-Anxiety Medications**

Some patients report **libido reduction or erectile changes**—which may be misinterpreted as worsening HFS.

**Common issues:**

- Difficulty obtaining an erection
- Reduced sensation
- Emotional blunting

However, others experience improvement because these meds reduce anxiety and sympathetic overactivation.

**F. Vacuum Pumps or Traction Devices**

These may irritate the neurovascular bundle if overused.

**Reported effects:**

- Numbness
- More shrinkage sensation
- Perineal tension from overcompensation

**G. Overuse of PDE5 inhibitors (Viagra, Cialis)**

Beneficial for some, but in others:

**Reported effects:**

- Pelvic congestion discomfort
- Penile “pressure” sensation
- Worsened nocturnal erections causing more guarding

**✅ 2. Why Some Treatments Make Symptoms Worse**

Hard flaccid syndrome often involves:

- Pelvic floor hypertonicity
- Sympathetic nervous system overactivation (“fight or flight”)
- Neurovascular irritation or entrapment
- Anxiety-loop reinforcement

Treatments that:

- **Increase muscle tension**,
- **Increase mechanical stress**, or
- **Increase anxiety regarding results**

may worsen the core dysfunction.

**✅ 3. Warning Signs a Treatment Is Too Aggressive**

If you are trying a therapy for HFS, stop or adjust it if you notice:

- Increasing flaccid firmness or shrinkage
- New burning or pressure sensations
- Worsening erectile quality
- More pelvic or perineal tightness
- New numbness or tingling

These are signs of **overstimulation or irritation**.

**✅ 4. Are These Worsenings Permanent?**

Almost always **no**.

Most reported worsenings:

- Are temporary (days to weeks)
- Improve when the irritating activity is stopped
- Resolve with relaxation-based therapy instead of strengthening

Patients rarely report permanent deterioration from treatments alone.

**✅ 5. Treatments Least Likely to Cause Worsening**

Based on clinical patterns and reports:

- **Diaphragmatic breathing**
- **Progressive relaxation**
- **Biofeedback** (down-training, not up-training)
- **Nerve gliding** (gentle only)
- **Mindfulness-based stress reduction**
- **Light aerobic exercise**
- **Manual PFPT (gentle, guided)**

These generally help without causing flares.

**📌 If you want, I can also provide:**

✔ A list of treatments with the **highest success rates**
✔ A personalized roadmap based on your symptoms
✔ A breakdown of why some people improve and others don’t
✔ A recovery timeline based on patient patterns

Just tell me what you’d like next.

# Symptoms and case presentation:

## Does hard flaccid syndrome affect my sexual and reproductive health?

Hard Flaccid Syndrome (HFS) can **affect sexual function**, but **it does *not* appear to cause permanent reproductive harm** based on what is currently known. Here is a clear breakdown:

**✅ Effect on Sexual Health**

**1. Erectile function**

Many people with HFS experience:

- Difficulty maintaining erections
- Erections that feel weaker or incomplete
- A “hard-but-soft” baseline flaccid state that makes sexual stimulation feel unusual

These symptoms are usually **functional**, often related to:

- Pelvic floor muscle dysfunction
- Nerve sensitization
- Anxiety or hypervigilance
- Vascular tension in the pelvic region

Importantly, these changes are **not due to structural damage to erectile tissue** in most cases.

**2. Libido (Sex Drive)**

HFS **does not directly affect hormone levels**, but stress, anxiety, and persistent symptoms may reduce sexual desire indirectly.

**3. Ejaculation**

Some people report:

- Delayed ejaculation
- Hypersensitivity or reduced sensation
- Pelvic floor tightness during or after orgasm

These are usually **functional symptoms** and typically improve when pelvic floor tension and anxiety improve.

**✅ Effect on Reproductive Health**

There is **no evidence that HFS causes:**

- Infertility
- Damage to the testes
- Reduced sperm count
- Hormonal dysfunction

Your ability to father children should **not** be affected by HFS.

**⚠️ When to be concerned (not necessarily HFS)**

Seek evaluation if you experience:

- Testicular swelling or severe pain
- Significant curvature or lumps in the penis
- Loss of sensation that continues to worsen
- Blood in semen
- Painful ejaculations that persist

These may be unrelated issues — such as prostatitis, nerve entrapment, or Peyronie’s disease — and deserve a proper exam.

**⭐ Summary**

**HFS mainly affects sexual function (erections, sensation, confidence), but not reproductive capacity.**
It is a **functional syndrome** driven by pelvic floor tension, autonomic imbalance, and stress — not by tissue destruction or irreversible damage.

If you want, I can also explain:

- Why HFS changes sexual sensations
- Which symptoms suggest pelvic floor involvement vs. nerve involvement
- What improves sexual function in HFS (evidence-based)

## What is the cause of the symptoms of hard flaccid syndrome?

Hard Flaccid Syndrome (HFS) is still not fully understood, but the **most supported explanations** involve a combination of pelvic floor dysfunction, vascular changes, and autonomic nervous system dysregulation. There is **no single proven cause**, but most patients share similar mechanisms.

Below is the clearest current understanding:

**✅ Most Likely Causes of Hard Flaccid Syndrome**

**1. Pelvic Floor Muscle Hypertonicity**

This is the **leading theory**.
Many patients develop tight or spasming pelvic floor muscles after:

- Excessive masturbation or sex
- Aggressive stretching / jelqing / vacuum pumps
- A minor penile or perineal injury
- Prolonged sitting or cycling
- Anxiety around performance or injury

When the pelvic floor becomes chronically tight:

- Blood flow to the penis can be reduced
- Nerves in the pelvic region become hypersensitive
- Erections may feel weaker or unstable
- The penis may appear firm but small when flaccid (the classic “hard-flaccid” state)

This creates a **self-reinforcing loop**: discomfort → muscle tension → worse symptoms.

**2. Neurovascular Irritation (Pudendal Nerve + Dorsal Penile Nerve)**

Many HFS symptoms match **pudendal or dorsal penile nerve irritation**:

- Numbness or reduced sensation
- Burning or tingling
- A feeling of fullness or pressure
- Changes in erection quality
- Pain after arousal or ejaculation

This can happen if muscles in the pelvic floor compress the nerve or if a prior minor injury caused localized inflammation.

**3. Dysregulation of the Autonomic Nervous System**

Stress, fear, and anxiety (including after the onset of symptoms) activate the **sympathetic nervous system** (“fight or flight”).

The sympathetic nervous system:

- Tightens pelvic floor muscles
- Reduces penile blood flow
- Interferes with erection sustainability
- Worsens penile sensitivity changes

Many patients report:

- Symptoms improve during deep relaxation
- Symptoms worsen during stress or sexual anxiety

This suggests an autonomic component, similar to chronic pelvic pain syndrome (CPPS).

**4. Venous Outflow Dysfunction / Microvascular Changes**

Some studies discuss local vascular dysregulation:

- Overactive pelvic floor muscles compress veins
- Blood cannot properly enter or leave the penis
- The flaccid penis becomes firm, cold, and small

This is not considered a primary cause, but a **secondary effect** made worse by muscle and nerve dysfunction.

**5. Psychosexual + Behavioral Triggers**

Not psychological *in origin*, but psychological factors can:

- Trigger the initial pelvic floor tension
- Maintain chronic tightness
- Increase vigilance toward genital sensations
- Create a cycle of symptom awareness → anxiety → worse symptoms

This is similar to how chronic back pain becomes persistent even after the initial injury heals.

**❌ What Does NOT Cause Hard Flaccid Syndrome**

There is *no evidence* that HFS is caused by:

- Testosterone deficiency
- Structural penile deformities
- Peyronie’s disease
- STI-related damage
- Permanent nerve destruction
- Irreversible vascular injury
- Infertility or testicular pathologies

HFS is **functional**, not a degenerative disease.

**🔍 Put Simply**

Hard flaccid syndrome is mostly caused by:

**Pelvic floor overactivation + nerve irritation + sympathetic overdrive**,
often triggered by a minor injury, sexual overuse, or stress.

This leads to:

- Flaccid firmness
- Loss of spontaneous erections
- Reduced sensitivity
- Erectile instability
- Pelvic/perineal tension
- Penile discomfort

**If you'd like**

I can also explain:

- How these mechanisms produce *specific symptoms*
- How to differentiate HFS from Peyronie’s, ED, CPPS, prostatitis, or nerve entrapment
- Evidence-based treatments (PFPT, trigger point release, autonomic down-training, nerve desensitization, lifestyle interventions)

Just tell me what you want next.

## Are the symptoms of hard flaccid syndrome dangerous? Can they be a sign of something worrisome?

Hard flaccid syndrome (HFS) can be very stressful and uncomfortable, but **the symptoms themselves are not usually dangerous or life-threatening**. However, they *can* overlap with or be triggered by other issues that may need medical evaluation. Here is a clear breakdown to help you understand what is and isn’t worrisome:

**✅ Are HFS symptoms dangerous by themselves?**

Generally, **no**.
HFS is considered a *functional* condition involving pelvic-floor muscle tension, neurovascular sensitivity, and sometimes psychological stress. The symptoms—such as a semi-firm flaccid penis, coldness, reduced sensation, urinary urgency, pain, or retraction—are distressing but **not known to cause long-term physical harm** or reproductive danger.

It does **not**:

- Damage the erectile tissue
- Cause infertility
- Lead to cancer
- Progressively worsen on its own once the underlying dysfunction is addressed

**🟡 But can HFS symptoms *signal* an underlying issue?**

**Sometimes, yes.** This is rare, but similar symptoms can be caused by other conditions that deserve medical attention.

**Conditions worth ruling out (not common)**

- **Pudendal nerve entrapment or irritation**
- **Pelvic floor hypertonicity** (most common contributing factor)
- **Chronic prostatitis / CPPS**
- **Vascular compression** (rare)
- **Low back issues** affecting nerve supply
- **Hormonal abnormalities** (less common)
- **Medication side effects**
- **Injury from aggressive sexual activity or exercise**

Most patients with symptoms matching HFS do *not* have a serious structural problem, but if symptoms began after:

- A direct trauma
- Intense masturbation or sex
- Heavy lifting / gym injury
- Sudden pelvic pain
  …then it’s worth having a clinician check things to rule out nerve or soft-tissue strain.

**🔴 Warning signs that require medical evaluation**

You should talk to a doctor if you notice:

- Progressive numbness spreading to the perineum or legs
- Difficulty controlling urination or bowel movements
- Persistent severe pelvic pain
- Erectile dysfunction that is rapidly worsening
- Visible testicular swelling, redness, or fever (signs of infection)
- Blood in urine or semen

These symptoms usually point to conditions *other than* HFS.

**🟢 When symptoms *do* match typical HFS**

If your symptoms are:

- Episodic changes in flaccid size/texture
- Pelvic tension
- Sensation changes
- Altered erections triggered by stress, anxiety, or overuse
- Normal function when distracted or relaxed
- Better in the daytime but worse at night or early morning

…then this strongly suggests a **functional pelvic-floor/nerve irritation pattern**, which is uncomfortable but **not dangerous**.

**⭐ Bottom line**

**Hard flaccid syndrome is not dangerous, but it is a real condition that can significantly impact quality of life.**
The symptoms usually reflect *reversible dysfunction*, not permanent damage.
But because similar symptoms can overlap with other pelvic or nerve conditions, a medical evaluation is appropriate if anything feels unusual, progressive, or severe.

If you want, you can tell me your specific symptoms and how they started, and I can help you understand whether they fit the typical HFS pattern or if anything stands out as concerning.

## Are there long-term side effects of hard flaccid syndrome?

Hard flaccid syndrome (HFS) is not known to cause **dangerous long-term physical damage**, but the condition **can** lead to longer-term effects if symptoms persist without improvement. Most of the long-term issues tend to be **functional, psychological, or quality-of-life related**, rather than structural or medically dangerous.

Here’s a clear breakdown:

**✅ Potential Long-Term Effects of Hard Flaccid Syndrome**

**1. Persistent pelvic floor dysfunction**

If the underlying problem is chronic pelvic floor muscle tension or dysregulation, symptoms may persist over time, including:

- A semi-rigid or tense flaccid penis
- Coldness or retraction
- Perineal discomfort or pressure
- Urinary or ejaculatory changes (usually mild)

These symptoms can wax and wane but typically **do not worsen progressively** like a degenerative disease.

**2. Sexual function issues (usually functional, not structural)**

Long-term symptoms may sometimes contribute to:

- Difficulty maintaining erections due to pelvic muscle overactivity or anxiety
- Increased sensitivity to arousal changes
- Delayed ejaculation or pelvic tightness during ejaculation
- Performance anxiety

**Important:**
HFS does **not** cause nerve death, hormonal problems, penile deformities, or testicular damage based on current evidence.

**3. Psychological impacts**

This is one of the most significant long-term effects:

- Health anxiety or hyperfocus on penile sensations
- Depression due to chronic symptoms
- Fear of long-term sexual dysfunction
- Sleep disturbance if symptoms worsen at night

These psychological cycles can reinforce pelvic floor tension, creating a feedback loop.

**4. Lifestyle limitations**

If untreated, some people adjust their routines to avoid symptom flares:

- Avoiding sex or dating
- Avoiding certain exercises (running, cycling, heavy lifting)
- Reduced physical activity due to discomfort
- Social withdrawal

**❗ What HFS does *not* typically cause long-term**

This is important for reassurance:

- No evidence of infertility
- No evidence of permanent penile tissue damage
- No progression into Peyronie’s disease
- No evidence of progressive nerve degeneration
- No effect on testosterone or reproductive organs

**⭐ Can long-term effects be prevented?**

Yes. Targeting the underlying pelvic-floor and neurovascular components early helps:

- Pelvic floor physical therapy
- Stress regulation + diaphragmatic breathing
- Avoiding repetitive trauma (e.g., aggressive masturbation, edging, hard kegels)
- Lifestyle adjustments (sleep, posture, exercise)
- Cognitive approaches for anxiety reduction

Many people experience partial or full recovery with time and proper management.

If you'd like, I can also help you with:

- **A list of treatments that have the best evidence**
- **What recovery timelines usually look like**
- **How to know if your symptoms are actually HFS or something else**
