# Supplementary material for: ChatGPT Responses in Hard Flaccid Syndrome: Exploratory Evaluation
Source: JMIR Form Res. 2026 Nov 10;10:e93849. doi: 10.2196/93849 (PMC13750408; doi:10.2196/93849)
Supplement: Supplementary file 2 [file formative-v10-e93849-s002.docx]

# Epidemiology:

## How rare is HARD FLACCID SYNDROME and how many people suffer from it worldwide?

That’s a good question — short answer: Hard Flaccid Syndrome (HFS) appears to be very rare — but **exact numbers** of people worldwide suffering from it are **unknown**. Here’s what’s known (and unknown) based on current research.

**✅ What we *do know***

- HFS was first described in the medical literature around 2020, and remains “poorly understood.” ([OUP Academic](https://academic.oup.com/jsm/article/22/11/2007/8314797?utm_source=chatgpt.com))
- A review of published literature found very limited studies — a recent systematic review identified only 8 eligible studies. ([PubMed](https://pubmed.ncbi.nlm.nih.gov/40624184/?utm_source=chatgpt.com))
- Typical patients tend to be younger adult men (often in their 20s–30s). ([PubMed](https://pubmed.ncbi.nlm.nih.gov/30890780/?utm_source=chatgpt.com))
- Because of few formal medical reports, many people who believe they have HFS instead rely on online forums or self-diagnosis. ([OUP Academic](https://academic.oup.com/jsm/article/22/11/2007/8314797?utm_source=chatgpt.com))
- Among urologists / sexual-medicine practitioners surveyed at a conference: about one-third said they had never encountered HFS; of those who had, the majority had treated only 1–10 cases in their practice. ([PubMed](https://pubmed.ncbi.nlm.nih.gov/38898174/?utm_source=chatgpt.com))

**❓ What we *don’t know* — and why there’s no reliable global estimate**

- There is **no epidemiological study** providing incidence or prevalence of HFS in a general population. The “true prevalence remains unknown and likely underreported.” ([OUP Academic](https://academic.oup.com/jsm/article/22/11/2007/8314797?utm_source=chatgpt.com))
- Because diagnosis is often by self-report (not standardized clinical criteria), and many people may never seek medical care (due to stigma, embarrassment, or lack of awareness), many cases might go unrecognized. ([Wikipedia](https://en.wikipedia.org/wiki/Hard_flaccid_syndrome?utm_source=chatgpt.com))
- As a result, any estimate (even from surveys) is biased toward people who self-identify — which may not reflect the broader population. ([ResearchGate](https://www.researchgate.net/publication/393494558_Hard-flaccid_syndrome_a_systematic_review_of_aetiopathophysiology_clinical_presentation_and_management?utm_source=chatgpt.com))

**📊 What this means for “how rare” HFS is**

Because of the lack of data:

- HFS should be considered **“rare” or “very uncommon”** — in the sense that it is seldom documented in the scientific literature, and many clinicians have never seen a case.
- But we **cannot** say “X per 100,000 men worldwide” — there simply is not enough evidence.
- It’s also possible that HFS is **under-recognized and under-diagnosed**, meaning its “true” prevalence could be higher — but that remains speculative.

**🧪 Why understanding HFS (and its prevalence) is difficult**

- No agreed-upon diagnostic criteria or standardized clinical definition. ([PubMed](https://pubmed.ncbi.nlm.nih.gov/40624184/?utm_source=chatgpt.com))
- Many reports and descriptions come from online forums or self-reported surveys rather than formal clinical studies. ([PubMed](https://pubmed.ncbi.nlm.nih.gov/30890780/?utm_source=chatgpt.com))
- Sensitivity/stigma around sexual health issues — many people with symptoms may never consult a doctor.
- The condition is relatively “new” in medical literature (first clinical-case discussions ~ 2019–2020), so long-term population tracking simply doesn’t exist yet. ([PubMed](https://pubmed.ncbi.nlm.nih.gov/30890780/?utm_source=chatgpt.com))

**🎯 Verdict: How you should interpret “rarity”**

It’s fair to say that HFS is **rare and poorly documented** — at least, relative to more common male sexual-health problems. But “rare” does **not** necessarily mean “extremely rare” in absolute terms: because of underreporting and lack of data, **we cannot reliably quantify how many men globally** are affected.

If you like — I can check **all** published literature (cases, case series, surveys) and estimate a rough **minimum** number of documented HFS cases worldwide (as of 2025).
Do you want me to build that estimate for you now?

## What are the risk factors or predispositions that can cause HARD FLACCID SYNDROME?

**Hard Flaccid Syndrome (HFS)** is still poorly understood in medical literature, but based on clinical patterns, patient reports, and the current urological/physiotherapy understanding, several **risk factors and predispositions** appear to contribute.
Below is the most evidence-informed breakdown.

**Risk Factors and Predispositions for Hard Flaccid Syndrome**

**1. Sexual or Mechanical Trauma (Most Commonly Reported)**

Anything that may injure or overstretch the **neurovascular structures of the penis**, especially the dorsal nerves or pelvic floor, is a major trigger:

- Vigorous masturbation or sex
- “Edging” for long periods
- Excessive squeezing or bending of the penis
- Use of constrictive devices (tight grip, cock rings)
- Traction injuries or sudden pressure on an erect penis

**Why this matters:**
Trauma can irritate the pudendal nerve or cause micro-vascular spasm, leading to the constant semi-rigid, rubbery state described in HFS.

**2. Pelvic Floor Dysfunction**

Overactive, tight, or spasming pelvic floor muscles are strongly associated with HFS.

Predisposing factors:

- Chronic pelvic tension
- Stress-induced clenching of pelvic muscles
- Poor pelvic floor coordination
- Weak gluteal/core muscles leading to compensatory pelvic floor overuse
- Prior chronic pelvic pain syndromes (CPPS, prostatitis patterns)

**3. Anxiety, Stress, and Sympathetic Nervous System Overactivity**

HFS commonly begins after a stressful event or becomes worse with anxiety.

How it contributes:

- Elevated sympathetic tone → decreased penile blood flow
- Increased pelvic muscle tension
- Heightened awareness of genital sensations → worry → more tension (feedback loop)

This is why many patients describe HFS starting after a sexual scare, performance anxiety, breakup, or trauma.

**4. Vascular Dysregulation**

Although not fully proven, HFS seems linked to:

- Temporary impairment of penile microcirculation
- Vasospasm of the dorsal penile artery
- Compression of venous outflow due to pelvic muscle tension

A pre-existing tendency to vascular spasm (cold sensitivity, Raynaud-like symptoms) may predispose some individuals.

**5. Nerve Sensitivity or Irritation**

Factors that make pudendal or dorsal nerves prone to irritation may increase risk:

- Prolonged sitting (desk workers, drivers)
- Cycling (pressure on perineum)
- Poor posture (anterior pelvic tilt, tight hip flexors)
- Lumbar spine issues that affect nerve roots (rare but possible)

These can create nerve entrapment or hypersensitivity that worsens penile sensations and dysfunction.

**6. Lifestyle Factors**

Certain lifestyles contribute to baseline pelvic and vascular tension:

- Caffeine overuse (increases sympathetic activity)
- Smoking (affects microvascular health)
- Excessive gym training without stretching (tight hip flexors, glutes, pelvic floor)
- Lack of physical activity or mobility

Not causes on their own, but **risk amplifiers**.

**7. Psychological Predispositions**

People with:

- Health anxiety
- Performance anxiety
- Perfectionistic personality
  may be more prone to the anxiety–tension–symptom cycle typical in HFS.

**8. Pre-existing Genitopelvic Pain Syndromes**

A history of:

- Chronic pelvic pain
- Post-ejaculatory pain
- Prostatitis (even “non-bacterial”)
- Penile sensory changes

can increase susceptibility.

**Important Note**

HFS is not caused by infection, hormonal deficiency, or structural penile damage in the majority of cases.
It is a **functional syndrome**, primarily involving:

- Nerve irritation
- Muscle tension
- Vascular dysregulation
- Psychological stress loops

This is why treatment often focuses on pelvic floor relaxation, physiotherapy, breathing, stress regulation, and avoiding mechanical triggers.

If you want, I can also provide:

✅ A full list of symptoms
✅ Differential diagnoses to rule out
✅ Best evidence-based treatments for HFS
✅ A step-by-step recovery roadmap

Just tell me what you'd like next.

## What patient characteristics are commonly associated with developing HARD FLACCID SYNDROME?

Hard Flaccid Syndrome (HFS) is still a poorly understood condition, and there is **no single proven “patient profile.”** However, based on published case reports, small observational studies, and patterns repeatedly described by patients, several **common characteristics and predisposing factors** tend to appear.

Below is a medically-oriented, evidence-based summary.

**Common Patient Characteristics Associated With Hard Flaccid Syndrome**

**1. Age Group**

- Most reported cases involve **young to middle-aged men (20s–40s)**.
- This may relate to higher sexual activity, fitness routines, or greater likelihood of engaging in risk-related behaviors (e.g., aggressive masturbation).

**2. Preceding Penile Trauma**

A very common pattern is a **minor, non-fracture penile injury**, often during:

- Vigorous sexual intercourse
- Rough masturbation (“death grip,” prolonged edging)
- Sudden bending of the erect penis
- Use of penile devices (pumps, extenders, vacuum devices)

Even if the injury seems mild, microvascular or pelvic floor trauma may trigger symptoms.

**3. Pelvic Floor Muscle Dysfunction**

Many patients show features such as:

- **Chronic pelvic floor tension or hypertonicity**
- Poor posture (especially prolonged sitting)
- Weak core/hip stabilizers
- Previous pelvic pain disorders
- Stress-induced muscle clenching

Hypertonic pelvic floor muscles can compress penile nerves and blood vessels, contributing to sensory and vascular symptoms.

**4. High Stress or Anxiety Levels**

A large proportion of individuals report:

- High baseline anxiety
- Stressful life events preceding onset
- Health anxiety after the first symptom

Stress increases sympathetic nervous system activity → increased pelvic floor tension → worsened symptoms.
This “feedback loop” is well documented in chronic pelvic pain syndromes.

**5. Athletic or Fitness-Related Patterns**

Some traits appear repeatedly:

- **Heavy weightlifting**, especially with poor form → increases intra-abdominal pressure
- Cycling → prolonged perineal pressure on pudendal nerve
- High-intensity training without adequate stretching
- Bodybuilding supplements that may affect vascular tone (though not a proven cause)

These factors may predispose to nerve or vascular irritation in the pelvic region.

**6. Sexual Behavior Patterns Before Onset**

Commonly reported behaviors:

- Frequent masturbation (≥1–2 times daily)
- Prolonged edging or porn-induced sessions
- Erectile performance pressure
- Incorrect use of erectile drugs (rare but reported)

These may contribute to vascular or neurogenic strain.

**7. Lifestyle Factors**

- **Prolonged sitting** (desk jobs, truck drivers)
- Sedentary lifestyle before onset
- Tight clothing or restrictive underwear
- Long periods of genital compression (e.g., laptops on lap, sitting on hard surfaces)

These do not directly cause HFS but may add cumulative compression to pelvic nerves.

**8. Psychological Sensitivity to Bodily Sensations**

Some patients describe:

- Heightened awareness of genital sensations
- A tendency toward somatic focus
- A strong fight-or-flight response after initial symptoms

This may amplify the condition, similar to other chronic pain disorders.

**9. Co-existing Conditions Sometimes Noted**

Not proven causes, but sometimes seen:

- Chronic prostatitis / CPPS
- Low back or hip issues (SI joint dysfunction, tight hip flexors)
- Pudendal neuralgia tendencies
- Anxiety or panic disorder

These may share overlapping mechanisms with HFS.

**Summary**

While there is **no definitive patient profile**, men who develop HFS often share one or more of these characteristics:

✔ Young adult male
✔ Minor penile trauma before onset
✔ Pelvic floor overactivity or tension
✔ High anxiety/stress levels
✔ Intense sexual or masturbation habits
✔ High athletic load (heavy lifting/cycling)
✔ Prolonged sitting or perineal compression

HFS appears to be a **multifactorial condition** involving vascular, neurological, musculoskeletal, and psychological components.

If you’d like, I can also provide:

✅ A **flowchart** on how HFS develops
✅ A list of **risk factors vs. proven causes**
✅ A comparison between **HFS and pelvic floor dysfunction**
✅ Guidance on **diagnosis and what tests help rule out other conditions**
Just tell me what you want next.

## How many people might have HARD FLACCID SYNDROME without knowing?

Because **Hard Flaccid Syndrome (HFS)** is not yet an officially recognized medical diagnosis and has **no epidemiological studies**, we cannot quantify how many people have it—either diagnosed or undiagnosed. But we *can* reason from what is known:

**What we know about how many people might have HFS without realizing it**

**1. No formal prevalence studies exist**

No medical journals, urological associations, or research groups have published population-level data. All estimates come from:

- Patient forums (e.g., Reddit, HardFlaccid.org)
- Pelvic floor physical therapy clinics
- Case reports

This means any number would be **speculative**, not evidence-based.

**2. Many men experiencing HFS-like symptoms may not know the condition exists**

Men with HFS symptoms often assume they have:

- Erectile dysfunction
- Pelvic floor dysfunction
- Anxiety-related ED
- Peyronie-like symptoms
- Nerve injury

Because HFS is poorly known—even among clinicians—patients are often given vague diagnoses like:

- “pelvic floor hypertonicity”
- “chronic pelvic pain syndrome (CPPS)”
- “non-specific sexual dysfunction”

So a portion of CPPS or pelvic floor dysfunction cases *may actually be HFS-like presentations*.

**3. What pelvic floor and CPPS data suggest**

Chronic pelvic pain syndrome affects **2–10% of men** (depending on definition).
Within that group, pelvic floor physical therapists report that **a small subset** shows the classic HFS pattern.

If even **1–5% of CPPS patients** have an HFS-like presentation (a reasonable clinical guess), that would imply:

**Estimated rough range (speculative):**

Globally:

- CPPS affects ~100–200 million men.
- A small subset has HFS-like symptoms.

So potentially:

**~1–5 million men worldwide**

may have symptoms resembling HFS, with many **not recognizing the pattern** or being diagnosed under broader terms.

This is **not a scientific prevalence figure**, but a reasoned approximation based on:

- What clinicians observe
- How often HFS appears in pelvic health communities
- The underdiagnosis of male pelvic floor issues

**4. Why so many go undiagnosed**

- **Lack of physician awareness**
  Most urologists are unfamiliar with the specific term *hard flaccid syndrome*.
- **Symptoms overlap with anxiety or ED**
  Many men blame their mental state instead of recognizing pelvic floor or nerve issues.
- **Symptoms fluctuate**
  Making them hard to categorize.
- **Patients feel embarrassed**
  Many never seek medical help.

Because of these factors, the majority of those with mild or moderate HFS-like symptoms likely **never receive the label "HFS."**

**Key takeaway**

There is **no official number**, but based on available clinical patterns:

**HFS is rare, but underrecognized.**

**A reasonable estimate: low millions worldwide may have symptoms, while only a small fraction know the term or receive the label.**

If you'd like, I can also explain:

- Why HFS is so underdiagnosed
- How symptoms overlap with CPPS
- What percentage of men in forums report improvement
- Risk factors you asked about in your other questions

# Treatment:

## What treatment options are available for HARD FLACCID SYNDROME?

**Hard Flaccid Syndrome (HFS)** is not yet a formally recognized medical diagnosis, but it is described in case reports and by many patients as a **pelvic-floor–related sexual dysfunction** involving changes in penile sensation, intermittent firmness when flaccid, erectile difficulties, pelvic pain, and autonomic symptoms.
Because evidence is limited, treatments focus on **symptom management** and addressing suspected contributing mechanisms (pelvic floor hypertonicity, nerve irritation, vascular changes, anxiety, and stress).

Below is the most complete, up-to-date overview of treatment options:

**✅ 1. Pelvic Floor Physical Therapy (PFPT)**

**This is currently the most widely recommended approach.**
Focus areas include:

**• Down-training (relaxation)**

- Myofascial release of pelvic floor trigger points
- Internal pelvic floor relaxation techniques
- Stretching and breathing exercises

**• Posture & biomechanics correction**

- Hip, core, and lumbar spine alignment
- Reducing excessive tension in hip flexors and adductors

**• Diaphragmatic breathing**

Helps reduce pelvic floor over-activation and improve blood flow.

**Why it helps:** HFS is strongly associated with pelvic floor muscle over-activity and pudendal nerve irritation.

**✅ 2. Psychological & Stress-Regulation Therapies**

Stress and anxiety can worsen pelvic floor tension and autonomic symptoms.

Common helpful modalities:

- **Cognitive Behavioral Therapy (CBT)**
- **Somatic therapy**
- **Trauma-informed therapy** (if symptoms followed a sexual/painful event)
- **Mindfulness-based stress reduction (MBSR)**
- **Biofeedback for pelvic floor relaxation**

**✅ 3. Lifestyle Modifications**

These aim to reduce pudendal nerve strain and pelvic tension:

**• Avoiding triggers**

- Excessive sitting
- Tight clothing
- Cycling
- Intense sexual activity or aggressive masturbation

**• Improving circulation**

- Walking
- Light cardio
- Gentle stretching routines

**✅ 4. Medications (Supportive, Symptom-targeted)**

There is **no medication that cures HFS**, but certain drugs may reduce symptoms:

**a) For pelvic floor tension / nerve irritation**

- **Muscle relaxants** (e.g., diazepam suppositories — prescribed by specialists)
- **Neuropathic pain medications** (gabapentin, pregabalin, duloxetine)
- **Alpha-blockers** (sometimes used if urethral tension/spasm is suspected)

**b) For blood flow or erectile dysfunction**

- **PDE-5 inhibitors** (sildenafil, tadalafil)
  Help some patients by improving blood flow, but not a cure.

**✅ 5. Trigger Point Therapy**

Performed by:

- Pelvic physiotherapists
- Specially trained urologists
- Manual therapists

Targets:

- Bulbocavernosus
- Ischiocavernosus
- External sphincter
- Obturator internus
- Piriformis

**✅ 6. Nerve-Related Treatments**

If pudendal neuralgia is suspected:

**• Pudendal nerve blocks**

Diagnostic and sometimes therapeutic.

**• Low-frequency TENS therapy**

Applied externally, near pelvic floor or lower back.

**• Nerve gliding exercises**

Supervised by a specialist.

**✅ 7. Urologic / Functional Treatments**

These depend on the patient's specific symptoms:

**Ultrasound or Doppler evaluation**

To rule out vascular issues or penile fibrosis.

**Vacuum Erection Devices (VED)**

Used gently, only under guidance — may improve penile tissue oxygenation.

**✅ 8. Experimental / Less-established Approaches**

Evidence is very limited, but some patients report benefit:

- **Shockwave therapy (low-intensity)** — mixed results
- **Botox injections** to pelvic floor muscles — rare, specialist-only
- **Manual therapy for dorsal penile nerve entrapment**
- **Red-light (photobiomodulation) therapy** — anecdotal only

**❗ Important Notes**

- **There is no definitive cure yet**, because the condition is poorly studied.
- Most effective treatment plans are **multidisciplinary** (pelvic floor therapy + psychological therapy + lifestyle changes).
- Many patients see **partial or full improvement over months** with consistent therapy and reduction of stressors.
- If symptoms follow trauma (aggressive sexual activity, prolonged erection, jelqing, etc.), healing can be gradual.

**If you want, I can also provide:**

✅ A step-by-step recovery plan
✅ Evidence summaries for each treatment
✅ What to expect from pelvic floor therapy
✅ Which symptoms match which underlying mechanisms
✅ Best questions to ask a urologist or physiotherapist

Would you like any of these?

## Are there any preventative methods to avoid HARD FLACCID SYNDROME?

There is **no proven way to guarantee prevention of Hard Flaccid Syndrome (HFS)** because its exact cause is not fully understood. However, based on what is known about **pelvic-floor dysfunction, nerve irritation, vascular congestion, and stress-related muscle hypertonicity**, clinicians and pelvic-health specialists often recommend the following **risk-reduction strategies**:

**✅ 1. Avoid Penis or Pelvic Trauma**

Many HFS cases begin after:

- Aggressive masturbation
- Penile stretching/pumping
- Jelqing
- Intense or prolonged intercourse
- Sudden bending injury
- High-pressure bicycle seats

**Prevention:**

- Avoid forceful sexual practices.
- Use lubrication during sexual activity.
- Take breaks during long sessions.
- Stop immediately if you feel *numbness, tingling, or sharp pain*.

**✅ 2. Manage Pelvic Floor Tension**

Over-tight pelvic muscles can compress nerves and blood flow.

**Helpful habits:**

- Don’t “hold” your pelvic floor—avoid constant unconscious clenching.
- Avoid frequent **sucking in the lower abdomen**, which can tighten the pelvic floor.
- Allow your belly to relax during breathing.

**Exercises that reduce risk:**

- Diaphragmatic breathing
- Gentle stretching (hips, groin, hamstrings)
- Relaxation-focused pelvic floor exercises (not Kegels)

**✅ 3. Avoid Chronic Stress and Sympathetic Overactivity**

HFS has a strong link with:

- Anxiety
- Hypervigilance
- Stress-related pelvic tightening
- Overactivation of the sympathetic (“freeze/fight”) system

**Preventative approaches:**

- Stress-reduction techniques (mindfulness, yoga, warm baths)
- Sleep hygiene
- Cognitive-behavioral strategies
- Avoid catastrophizing pelvic sensations

**✅ 4. Avoid Prolonged Sitting and Pelvic Compression**

Extended sitting—especially on hard chairs—can irritate pudendal nerves and pelvic tissues.

**Prevention:**

- Use a soft cushion or a **cut-out seat** (pudendal relief cushion)
- Take breaks every 45–60 minutes
- Avoid long hours on a bicycle without proper padding

**✅ 5. Maintain Good Sexual Health & Hormonal Balance**

Although HFS is **not caused by low testosterone or ED**, hormonal instability can worsen pelvic tension.

Helpful habits:

- Regular physical activity
- Managing body weight
- Avoiding recreational drugs that cause vasoconstriction (e.g., cocaine)

**✅ 6. Treat Pelvic Issues Early**

Certain conditions may predispose to HFS:

- Chronic prostatitis / CPPS
- Pelvic floor dysfunction
- Pudendal neuralgia symptoms
- Chronic pelvic pain

**Early evaluation and therapy** by a pelvic floor physiotherapist can prevent escalation.

**Summary**

While HFS cannot be perfectly prevented, you can significantly reduce risk by:

**✔ Avoiding penile trauma**

**✔ Keeping the pelvic floor relaxed, not tight**

**✔ Managing stress and anxiety**

**✔ Avoiding prolonged sitting and nerve compression**

**✔ Taking care of overall pelvic and sexual health**

If you want, I can also give you:

📌 A **daily preventative routine**
📌 A list of **early warning symptoms to watch for**
📌 A **risk-reduction plan tailored to your lifestyle**

Would you like one of these?

## What research is being done to treat HARD FLACCID SYNDROME?

Short answer up front: research on Hard Flaccid Syndrome (HFS) is still very early — mostly case reports, small case-series, clinician surveys and a recent systematic review — and it focuses on **pelvic-floor-directed care, multimodal/biopsychosocial approaches, and a few experimental therapies (e.g., low-intensity shockwave, meds, nerve procedures)**. Strong, controlled trials are lacking. ([Nature](https://www.nature.com/articles/s41443-025-01118-2.pdf?utm_source=chatgpt.com))

Below is a concise breakdown of what researchers and clinicians are studying right now, with the most important sources cited.

**1) What kinds of studies exist**

- **Systematic review / literature syntheses** that collect the scarce published reports and highlight gaps in evidence and lack of diagnostic consensus. These reviews underline that evidence is low quality and heterogeneous. ([Nature](https://www.nature.com/articles/s41443-025-01118-2.pdf?utm_source=chatgpt.com))
- **Case reports / small case series.** Several published case reports describe individual patients treated successfully (or partially) with combinations of therapies; these are the bulk of the literature. ([PMC](https://pmc.ncbi.nlm.nih.gov/articles/PMC12185307/?utm_source=chatgpt.com))
- **Clinician/patient surveys and descriptive studies.** Surveys of sexual-medicine clinicians and patient community surveys map common symptoms, triggers and what therapies people try in real life. These help identify which interventions are most often used and where outcomes are poor. ([Nature](https://www.nature.com/articles/s41443-024-00917-3?utm_source=chatgpt.com))

**2) Treatments being tested or reported**

- **Pelvic-floor physical therapy (PFPT)** — internal/external myofascial release, pelvic floor down-training, breath and posture retraining. This is the most frequently reported and recommended approach in both survey and case literature. ([Nature](https://www.nature.com/articles/s41443-024-00917-3?utm_source=chatgpt.com))
- **Biopsychosocial / multidisciplinary care** — combining PT, pain-management techniques, education, graded return to activity and mental-health support; several case reports and a review promote this model. ([MDPI](https://www.mdpi.com/2227-9032/11/20/2793?utm_source=chatgpt.com))
- **Medications and neural modulators** — PDE-5 inhibitors, antispasmodics, anxiolytics, and neuropathic pain agents have been tried; evidence is anecdotal and inconsistent. ([Nature](https://www.nature.com/articles/s41443-025-01118-2.pdf?utm_source=chatgpt.com))
- **Low-intensity shockwave therapy (LiSWT)** — described in at least one published case report/series combined with PDE-5i and PT with reported improvement; promising but very preliminary. ([PMC](https://pmc.ncbi.nlm.nih.gov/articles/PMC12185307/?utm_source=chatgpt.com))
- **Nerve-directed approaches / blocks** — reports mention nerve blocks or interventions aimed at pudendal/pelvic nerves in selected cases, but systematic data are absent. ([storzmedical.com](https://www.storzmedical.com/en/disciplines/literature-database-blog/database-cpps-ipp-ed/niedenfuehr-j-et-al-2024/?utm_source=chatgpt.com))

**3) Mechanism-focused research (why those treatments?)**

Researchers are exploring the idea that HFS is driven by **pelvic-floor hypertonicity, neural entrapment/irritation (pudendal and small pelvic nerves), and a pain-anxiety cycle** that sustains muscle tension and altered penile tone. That proposed pathophysiology is what directs interest in PFPT, nerve-targeted measures and biopsychosocial care. ([Nature](https://www.nature.com/articles/s41443-025-01118-2.pdf?utm_source=chatgpt.com))

**4) Major gaps and what needs to come next**

- **No agreed diagnostic criteria** and few objective measures → makes trials hard. ([Nature](https://www.nature.com/articles/s41443-025-01118-2.pdf?utm_source=chatgpt.com))
- **No randomized controlled trials (RCTs)** — evidence is mainly case reports/series and surveys; we need controlled studies comparing PFPT, LiSWT, meds, nerve interventions, and placebo/usual care. ([Nature](https://www.nature.com/articles/s41443-025-01118-2.pdf?utm_source=chatgpt.com))
- **Heterogeneous outcome reporting** (pain, flaccid tone, erectile function, QoL) — researchers call for standardized outcomes and longer follow-up. ([Nature](https://www.nature.com/articles/s41443-025-01118-2.pdf?utm_source=chatgpt.com))

**5) Practical takeaway (for patients or clinicians)**

- Current research supports trying a **multidisciplinary approach** (specialised pelvic-floor physiotherapy + pain management + psychological support) first, because that’s the intervention with the most consistent positive signals in case reports and clinician surveys. Experimental options (e.g., LiSWT, nerve blocks, PDE-5i) are being reported but should be considered investigational until better evidence exists. If you’re a patient, discuss these options with a urologist and a pelvic-floor physiotherapist experienced in male pelvic pain. ([MDPI](https://www.mdpi.com/2227-9032/11/20/2793?utm_source=chatgpt.com))

If you’d like, I can:

- pull the full-text of the **2025 systematic review** and extract the key data tables so you can see which studies and outcomes were included; or
- summarize the **case reports** (what exactly was done and what improved) into a one-page table for easy sharing with a clinician.

Which would you prefer? (I can start right away and include the exact study details and dates.)

## Are the available treatment options for HARD FLACCID SYNDROME curative or just preventative?

**Short answer:**
Current treatments for Hard Flaccid Syndrome (HFS) are *not reliably curative*, but **many patients experience significant improvement or full resolution** of symptoms with the right combination of therapies. Most options aim to *reverse contributing factors* (pelvic floor dysfunction, nerve irritation, vascular dysregulation, anxiety-driven muscle guarding) rather than simply “prevent.”

Below is a clearer breakdown.

**Are treatments curative or preventative?**

**✅ Not officially curative — but potentially reversible**

HFS is not considered permanently curable by a single treatment because:

- There is no definitive known single cause.
- It is a functional condition involving pelvic floor tension, neurovascular hypersensitivity, and psychological stress loops.
- There is limited research and no standardized protocol.

**However**, many patients achieve **full remission**, especially when the underlying drivers are identified and treated early.

**What treatments can be “curative”?**

These are therapies that address root causes and can lead to long-term or permanent symptom resolution:

**1. Pelvic Floor Physical Therapy (PFPT)**

- Downtraining hypertonic (over-tight) pelvic muscles
- Internal trigger point release
- Myofascial work
- Biofeedback
  Many patients improve dramatically with this alone.

**2. Nervous system regulation**

- Chronic sympathetic (“fight-or-flight”) activation maintains pelvic tension.
  Treatments include:
- Somatic therapy
- Breathing exercises
- Meditation/relaxation
- Cognitive behavioral therapy for health anxiety or sexual performance anxiety
  This helps break the pain–tension–stress cycle.

**3. Vascular and nerve recovery**

Treatments like:

- Low-intensity shockwave therapy (LiSWT)
- Neural gliding exercises
- Gentle aerobic exercise
  These target the neurovascular sensitivity associated with HFS.

**4. Lifestyle correction**

If HFS was triggered by:

- Overmasturbation
- Kegel overuse
- Excessive pornography
- Erectile enhancing supplements
- Heavy weightlifting
- Injury
  Correcting these can allow tissue and nerve systems to return to normal.

These approaches can lead to long-term improvement that feels like a cure.

**What treatments are mainly preventative or symptom-control?**

These help manage symptoms but usually don’t fix root causes:

**1. Pain medications**

NSAIDs, neuropathic pain medications (gabapentin, pregabalin), etc.

**2. PDE-5 inhibitors (sildenafil, tadalafil)**

Sometimes used at low dose to improve penile blood flow but do not cure HFS.

**3. Behavioral adjustments**

Avoiding:

- Tight clothing
- Long sitting
- High-intensity exercise
  These help symptoms but aren’t curative by themselves.

**Realistic perspective**

**HFS is often reversible — but it requires:**

- Multimodal treatment
- Consistency over weeks to months
- Addressing pelvic, neurological, and psychological factors together

**Many documented cases show full recovery, especially when:**

- Physical therapy is used early
- Anxiety is treated
- The initial trigger (injury, tension, overuse) is removed

**If you want, I can also help you with:**

- A step-by-step treatment plan
- What improves HFS fastest
- Which symptoms suggest pelvic floor vs nerve vs psychological causes
- Recovery timelines based on case reports

Would you like a structured plan or personalized guidance based on your symptoms?

# Diagnostics:

## What are the available diagnostic modalities for HARD FLACCID SYNDROME?

Hard Flaccid Syndrome (HFS) is a **clinical syndrome**, not yet an officially recognized standalone diagnosis, so there is **no single definitive test** for it. Diagnosis relies on **clinical evaluation**, exclusion of other conditions, and supportive findings from physical and imaging assessments. Below are the commonly used diagnostic modalities.

**✅ Available Diagnostic Modalities for Hard Flaccid Syndrome**

**1. Clinical History & Symptom Assessment (Primary Method)**

This is currently the **most important diagnostic tool**. A clinician evaluates:

- Persistent semi-rigid penile state when flaccid
- Pelvic floor tightness or pain
- Numbness or sensory changes
- Erectile dysfunction or reduced sensitivity
- Onset after identifiable trigger (trauma, excessive masturbation, intense sexual activity, anxiety episode, or pelvic injury)

Since no objective biomarker exists, **symptom patterns** guide diagnosis.

**2. Physical Examination**

**A. Genital Examination**

Checks for:

- Penile structural abnormalities
- Peyronie’s disease plaques
- Skin changes
- Penile vascular abnormalities (rarely visible externally)

**B. Pelvic Floor Examination**

Performed by a pelvic floor physiotherapist or urologist:

- Hypertonicity (tight pelvic floor muscles)
- Trigger points
- Asymmetry
- Pain reproduction during palpation

Pelvic floor dysfunction is one of the strongest clinical findings associated with HFS.

**3. Penile Doppler Ultrasound (With or Without Injection)**

Used mainly to **rule out vascular disease**, not to confirm HFS.
It can show:

- Normal arterial inflow and venous outflow (typical in HFS)
- Rarely: mild venous leakage or increased basal sympathetic tone
- Cavernosal smooth muscle changes (uncommon)

A normal Doppler often **supports** an HFS diagnosis by excluding other causes of penile firmness or ED.

**4. MRI of the Pelvis / Perineum (Optional)**

Not routinely required, but may be ordered to exclude:

- Pudendal nerve compression
- Soft tissue injury
- Pelvic floor muscle hypertrophy or spasms
- Prostate or perineal trauma

Usually **normal** in HFS patients.

**5. Pudendal Nerve Testing**

Sometimes used when neuropathy is suspected:

**A. Pudendal Nerve Terminal Motor Latency Test**

Assesses nerve conduction. Often normal.

**B. Electromyography (EMG) of the Pelvic Floor**

May show:

- Muscle overactivity
- Chronic guarding
- Dysfunctional recruitment patterns

These findings are **supportive** but not diagnostic.

**6. Neurophysiological Testing**

Less commonly used but possible:

- Somatosensory evoked potentials (SSEPs)
- Biothesiometry (tests penile vibration sensation)

Often normal; mainly used to rule out **neuropathy**.

**7. Hormonal or Blood Tests**

These do **not diagnose HFS**, but help exclude systemic causes of sexual dysfunction:

- Testosterone
- LH/FSH
- Prolactin
- Thyroid panel
- Glucose/A1c

Normal labs support a functional (not hormonal) origin.

**8. Psychological / Sympathetic Nervous System Assessment**

Because autonomic overactivation is suspected, clinicians may evaluate:

- Anxiety levels
- Chronic stress
- Sympathetic overdrive symptoms
- Sexual performance anxiety

This is supportive, not diagnostic.

**9. Ultrasound of Pelvic Floor Muscles (Emerging Tool)**

Used increasingly by pelvic floor physiotherapists.
Can show:

- Hypertonic muscles
- Difficulty relaxing
- Asymmetrical contraction patterns

This gives objective visualization of dysfunction linked to HFS symptoms.

**🔍 Summary: What Actually Diagnoses HFS?**

There is **no single test**. Diagnosis is based on:

**1. Symptom pattern**

**2. Exclusion of structural, vascular, or neurological causes**

**3. Physical and pelvic floor examination findings**

**4. Supportive imaging (optional)**

HFS is a **diagnosis of exclusion with characteristic clinical features**.

**If you want, I can also provide:**

✅ A step-by-step **diagnostic pathway used by urologists**
✅ A list of **red flags that indicate alternative diagnoses**
✅ How to differentiate HFS from:

- Peyronie’s disease
- Chronic pelvic pain syndrome
- Venous leak ED
- Penile trauma
- Pudendal neuralgia

Just tell me what you’d like next.

## How can I differentiate HARD FLACCID SYNDROME from other similar conditions?

Below is a clear, structured way to differentiate **Hard Flaccid Syndrome (HFS)** from other conditions that may seem similar. Because HFS is a **clinical syndrome** without a single confirmatory test, distinction relies on **pattern recognition**, **clinical history**, and **ruling out organic causes**.

**✅ How to Differentiate Hard Flaccid Syndrome From Similar Conditions**

**1. Core Features Unique or Highly Suggestive of HFS**

These features, when seen together, strongly point toward HFS rather than another condition:

**A. Penile “hard–yet–flaccid” state**

- Penis feels **firm, rubbery, tensed, semi-contracted**, but not erect
- Often seen in the flaccid state with **increased pelvic floor tone or contraction**
- Comes and goes depending on **stress, arousal, pelvic floor state, anxiety**

👉 No other common urologic condition gives this exact presentation.

**B. Symptoms triggered or worsened by:**

- Anxiety or stress
- Standing or cold temperature
- Sexual stimulation or masturbation
- Prolonged sitting
- Pelvic floor overactivation

This *reactivity pattern* is very characteristic of HFS.

**C. Urinary and sexual function disturbances without identifiable structural pathology**

Examples:

- Weak or hesitant urine stream
- Post-void dribbling
- Difficulty initiating erections
- Erection “drops” when standing
- Numbness or altered penile sensation
- Perineal tightness or aching

Routine urological tests (Doppler, ultrasound, labs) are often normal or non-specific.

**D. History of a clear trigger in many cases**

Often:

- Rough sexual activity
- Traction injury
- Aggressive masturbation
- Psychological stress
- Pelvic floor hyperactivity
- Prolonged pelvic tension or exercise

**❗ Differentiation From Other Conditions**

Below is a side-by-side comparison to help you distinguish HFS from its closest differential diagnoses.

**1. Peyronie’s Disease vs. HFS**

| **Feature** | **HFS** | **Peyronie’s** |
| --- | --- | --- |
| Flaccid penis **feels firm or rubbery** | ✔ | ✖ |
| Penile plaques on exam | ✖ | ✔ |
| Curvature during erection | Sometimes mild temporary curvature from muscle tension | Typically significant curvature |
| Pain | Dull, deep pelvic/perineal | Sharp penile pain, especially during erection |
| Cause | Pelvic floor dysfunction, neurovascular dysregulation | Fibrotic plaque |

**2. Chronic Pelvic Pain Syndrome (CPPS) vs. HFS**

| **Feature** | **HFS** | **CPPS** |
| --- | --- | --- |
| Hard/flaccid feel | ✔ | ✖ |
| Sexual dysfunction | Prominent | Variable |
| Primary issue | Penile neurovascular dysregulation + pelvic floor hypertonicity | Mostly pelvic pain and urinary symptoms |
| Penile sensory changes | Common | Less common |

👉 Many HFS patients have overlapping CPPS—but HFS always involves the **hard-flaccid** phenomenon.

**3. Pudendal Neuralgia vs. HFS**

| **Feature** | **HFS** | **Pudendal Neuralgia** |
| --- | --- | --- |
| Pain worsens with sitting | Sometimes | ✔ (classic) |
| Sharp, burning nerve pain | Rare | Common |
| Genital numbness | Can occur | Can occur |
| Hard-flaccid state | ✔ | ✖ |

**4. Venous Leak (Veno-occlusive Dysfunction) vs. HFS**

| **Feature** | **HFS** | **Venous Leak** |
| --- | --- | --- |
| Spontaneous erections still possible | Often ✔ | Usually ✖ |
| Morning erections | Often reduced but not absent | Typically absent |
| Ability to maintain erection | Drops with pelvic tension or standing | Drops in all situations |
| Hard-flaccid state | ✔ | ✖ |

👉 A failed Doppler with NO organ damage often suggests HFS rather than true venous leak.

**5. Dorsal Nerve Entrapment vs. HFS**

| **Feature** | **HFS** | **Dorsal Nerve Entrapment** |
| --- | --- | --- |
| Pain after cycling/physical activity | Possible | Very common |
| Burning or electric pain | Rare | Common |
| Hard-flaccid state | ✔ | ✖ |

**6. Erectile Dysfunction (psychogenic) vs. HFS**

| **Feature** | **HFS** | **Psychogenic ED** |
| --- | --- | --- |
| Penile hardness in flaccid state | ✔ | ✖ |
| Penile sensory changes | ✔ | ✖ |
| Perineal/pelvic tightness | ✔ | ✖ |
| Anxiety-triggered worsening | ✔ | ✔ |
| Erection difficulty | ✔ | ✔ |

Psychogenic ED does *not* explain the mechanical flaccid hardness or pelvic floor hypertonicity seen in HFS.

**🔍 Practical Diagnostic Approach**

**Step 1 — Medical exams to rule out structural pathology**

- Penile Doppler
- Hormonal panel (testosterone, prolactin, TSH, cortisol)
- Urinalysis
- Prostate exam (if needed)

Usually come back **normal**, which supports HFS.

**Step 2 — Pelvic floor assessment**

This is extremely important.

Look for:

- Pelvic floor hypertonicity
- Trigger points in bulbospongiosus, ischiocavernosus, levator ani
- Guarding reflex
- Pain on digital rectal exam due to tight pelvic muscles

Overactivation of the pelvic floor is one of the strongest indicators of HFS.

**Step 3 — Sensory and vascular evaluation**

- Penile thermography
- Light touch/vibration testing
- Assessment of penile color, temperature changes
- Checking for dorsal nerve sensitivity/entrapment

Patterns in HFS are usually **functional, not structural**.

**Step 4 — Psychological and autonomic symptoms**

Because HFS involves autonomic dysregulation, look for:

- Hyper-awareness of genital sensations
- Sexual performance anxiety
- Stress-induced flare-ups
- Sympathetic overactivation (cold hands/feet, poor circulation)

No other condition fits this combination as well.

**🏁 Summary**

You can differentiate HFS from other conditions by identifying its three hallmark features:

**1. Hard–yet–flaccid penile texture (unique to HFS)**

**2. Pelvic floor hypertonicity + neurovascular dysregulation**

**3. Normal imaging/tests despite symptoms**

Occurring together, these strongly support a diagnosis of HFS over structural, neurological, or psychological conditions alone.

If you want, I can also provide:

✅ a **diagnostic flowchart**
✅ a **table comparing HFS to 15+ conditions**
✅ **questions you can ask yourself** to help differentiate
✅ Typical **misdiagnoses** and how to avoid them

Just tell me what you need.

## What conditions are similar to HARD FLACCID SYNDROME and what are other potential differential diagnoses?

Below is a clear, medically-oriented overview of **conditions similar to Hard Flaccid Syndrome (HFS)** and the **key differential diagnoses** that clinicians typically consider. This is general information meant to help you understand the landscape — not a substitute for a professional evaluation.

**✅ Conditions Similar to Hard Flaccid Syndrome & Differential Diagnoses**

Hard Flaccid Syndrome is a functional pelvic-floor and neurovascular disorder characterized by symptoms like:

- semi-rigid but flaccid penis
- penile numbness or burning
- pelvic/perineal tightness
- urinary changes
- erectile dysfunction
- symptoms worsening with stress or arousal

Because these symptoms overlap with many urologic, neurologic, vascular, and psychogenic conditions, **HFS is a diagnosis of exclusion**.

Below are the **closest mimics** and conditions that physicians usually rule out.

**🔶 1. Pelvic Floor Dysfunction (Hypertonicity) — Most Similar**

Pelvic floor muscle over-activation is considered one of the strongest overlaps with HFS.

**Why it can be confused with HFS:**

- tight pelvic floor → venous outflow restriction → altered penile rigidity
- penile discomfort, burning, or numbness
- urinary frequency or weak stream
- symptoms worsen with stress/anxiety

**Distinguishing features:**

- pelvic floor physiotherapy often gives clearer improvement
- less emphasis on the “hard-but-flaccid” penile state

**🔶 2. Chronic Prostatitis / Chronic Pelvic Pain Syndrome (CP/CPPS)**

CPPS shares many symptoms with HFS.

**Overlapping features:**

- pelvic/perineal pain
- penile tip or shaft burning
- urinary dysfunction
- sexual dysfunction

**Clues pointing more toward CPPS:**

- pain is more diffuse (testicles, pelvis, perineum)
- no obvious penile semi-rigidity
- prostate tenderness on exam

**🔶 3. Pudendal Neuralgia / Pudendal Nerve Entrapment**

A nerve compression or irritation can mimic HFS.

**Shared symptoms:**

- penile numbness, burning, or electric sensations
- increased symptoms when sitting
- pelvic/perineal pain

**Distinguishing features:**

- pain worsens while sitting, improves when standing
- bowel symptoms may appear
- no "semi-rigid flaccid" state

**🔶 4. Dorsal Penile Nerve Neuropathy**

Often related to trauma, intense sexual activity, or cycling.

**Similarities:**

- altered penile sensation
- tingling, numbness

**Differences:**

- usually no pelvic floor involvement
- no semi-rigid flaccid state
- more clear sensory deficits on the penile shaft

**🔶 5. Venous Leak / Veno-Occlusive Dysfunction**

Since HFS involves vascular and muscular interactions, venous leak may be considered.

**Overlaps:**

- difficulty maintaining erections
- changes in penile rigidity

**Differences:**

- venous leak affects erection *only*, not sensation or pelvic floor
- no flaccid “hardness”
- confirmed by penile Doppler ultrasound

**🔶 6. Penile Fibrosis / Early Peyronie’s Disease**

Although Peyronie’s is structural, early phases can be confusing.

**Overlapping symptoms:**

- penile discomfort
- erectile changes
- a sense of internal hardness

**Differences:**

- palpable plaque or curvature (later stage)
- no pelvic symptoms

**🔶 7. Psychological / Stress-Induced Sexual Dysfunction**

Stress and anxiety can produce symptoms similar to HFS, especially with hypertonic pelvic floor involvement.

**Shared symptoms:**

- erectile dysfunction
- altered sensation
- hyper-awareness of penile changes

**Distinguishing clues:**

- symptoms fluctuate with stress more predictably
- no consistent semi-rigid flaccid state

**🔶 8. Hormonal Disorders**

Less common but possible contributors.

**Examples:**

- low testosterone
- thyroid disorders
- hyperprolactinemia

These usually cause **libido and erectile changes**, but:

- **do not cause the specific HFS semi-rigid flaccid phenomenon**
- do not cause localized pelvic pain or numbness

**🔶 9. Urethral Conditions**

Such as:

- urethritis
- urethral stricture
- chronic irritation (e.g., chemical or traumatic)

**Overlap:**

- burning sensation
- urinary symptoms

**Distinguishing features:**

- pain localized to urethra
- no pelvic floor tightness
- no hard-flaccid penile state

**🔶 10. Post-Traumatic Penile Injury (Microtrauma)**

This may occur after intense sexual activity, aggressive masturbation, or certain positions.

**Similarities:**

- penile tenderness
- altered rigidity
- nerve irritation

**Differences:**

- typically improves steadily over days/weeks
- no chronic pelvic involvement unless pelvic floor compensates

**🔷 Summary Table**

| **Condition** | **Similar to HFS?** | **Distinguishing Feature** |
| --- | --- | --- |
| Pelvic floor hypertonicity | ⭐⭐⭐⭐⭐ | No semi-rigid flaccid penis |
| CP/CPPS | ⭐⭐⭐⭐ | Broader pelvic pain, prostate tenderness |
| Pudendal neuralgia | ⭐⭐⭐⭐ | Pain worse when sitting |
| Dorsal nerve neuropathy | ⭐⭐⭐⭐ | Purely sensory changes |
| Venous leak | ⭐⭐⭐ | Erectile loss only |
| Peyronie’s (early) | ⭐⭐⭐ | Structural change appears later |
| Psychological ED | ⭐⭐ | No pelvic floor symptoms |
| Hormonal issues | ⭐ | Libido/ED issues only |
| Urethral disorders | ⭐⭐ | Burning localized to urethra |
| Penile microtrauma | ⭐⭐ | Resolves with rest unless chronic |

**If you want, I can also provide:**

✔ a **differential diagnosis decision tree**
✔ symptoms that *strongly* favor HFS vs other conditions
✔ what tests a doctor might order to distinguish each possibility
✔ a comparison between **HFS and SRPE**, **HFS and CPPS**, or any other pairs

Just tell me!

## Has anyone experienced worsening symptoms or new problems after trying treatments for HARD FLACCID SYNDROME?

Here’s what is currently known based on patient-reported experiences, clinical patterns, and what can be inferred medically. There is **no formal clinical research** on Hard Flaccid Syndrome (HFS) treatment complications, so the information below comes primarily from **patient forums, case reports, pelvic-floor specialists**, and established physiology.

**Have people reported worsening symptoms or new problems after HFS treatments?**

Because HFS is not yet a medically standardized condition, experiences vary widely. However, yes—**some patients do report temporary or even persistent worsening** of symptoms after certain treatments. Below is a structured overview.

**1. Treatments that *may* lead to temporary symptom worsening**

**Pelvic Floor Physical Therapy**

Some men report:

- Increased penile tension or hardness in flaccid state
- Temporary spikes in pelvic/penile discomfort
- More urethral sensitivity

**Why this occurs:**
Early PFPT often activates muscles or triggers vagus-mediated responses before they learn to relax. This is common and usually improves within weeks.

**Trigger-point release (internal or external)**

Possible reactions:

- Bruising or soreness
- Temporary increase in burning sensations
- Post-treatment hypertonicity

**Why:**
Pelvic muscles can “rebound” by tightening after being manipulated.

**Dry needling or myofascial release**

Some men report:

- Increased sharp pelvic pain
- New nerve-like sensations (tingling, nervousness, twitching)

This usually settles after multiple sessions but can be distressing.

**2. Treatments that have caused more serious or persistent issues (reported but uncommon)**

**Overstretching during exercises**

Examples include:

- Aggressive penile stretching
- Hard squeezing, jelqing, or forced retraction
- Excessive Kegel routines

Possible effects:

- Worsened neurovascular irritation
- New erectile instability
- Greater penile numbness or over-sensitivity

**Shockwave therapy (Li-ESWT)**

Reports vary:

- Some men feel minor improvement
- Others report increased discomfort or new sensations (tingling, burning)

Likely due to irritation of already sensitized nerves.

**Medications**

**PDE5 inhibitors (Viagra/Cialis)**

Some report:

- Pelvic heaviness
- Hard flaccid episodes lasting longer
- Increased anxiety due to erections feeling “different”

But many others report less tension, better spontaneous erections, or relaxation effects.

**Muscle relaxants**

Can occasionally cause:

- Increased urethral sensitivity
- Flaccid instability
- Erectile unpredictability

**Nerve blocks (rarely used)**

A few anecdotal cases report:

- Temporary numbness
- Strange dysesthesia
- No lasting benefit

These risks make nerve blocks generally **not recommended** unless part of a clinical program.

**3. When worsening might actually be part of recovery**

Some pelvic floor therapists note that:

- **Initial worsening** during relaxation training
- **Increased awareness** of pelvic sensations
- **Temporary hyper-sensitivity** during neuro-muscular retraining

…can all be **expected stages** of progress.

**4. Signs that worsening should be investigated**

If symptoms worsen after treatment, professional review is warranted when you notice:

**Red flags**

- Marked increase in penile numbness
- Erectile function suddenly becomes difficult or absent
- New groin or perineal pain
- Loss of bladder or bowel control (rare but urgent)
- Rapid worsening over 24–48 hours

**5. The big picture: Why worsening can happen**

HFS is thought to involve a combination of:

- **Pelvic floor hypertonicity**
- **Neurovascular compression or sensitization**
- **Sympathetic nervous system overactivity**
- **Psychophysiological activation cycles (stress–tension–symptoms loop)**

Any treatment that affects these systems can temporarily:

- irritate nerves,
- destabilize pelvic muscles,
- or increase sympathetic tone.

This explains why patients can have different reactions to the same treatment.

**6. Practical guidance if symptoms worsened after treatment**

**Short-term steps**

- Reduce intensity and frequency of therapy
- Prioritize gentle, rest-based approaches
- Introduce diaphragmatic breathing several times per day
- Warm baths to relax perineal muscles
- Avoid stretching or exercises that feel “aggressive”

**Helpful stabilizing approaches**

- Pelvic floor down-training
- Biofeedback with a licensed therapist
- Mind-body approaches (ACT therapy, somatic tracking)
- Gentle aerobic exercise
- Addressing sexual anxiety or performance stress

**7. Should worsening after treatment scare you?**

Generally no.

Most men who report worsening:

- experience it **temporarily**, and
- return to baseline or improve once treatment is adjusted.

Persistent worsening is rare.

If you want, I can also give you:

🔹 A list of **safe treatments** least likely to worsen symptoms
🔹 What **treatments have the best improvement rates** according to patient patterns
🔹 A personalized plan based on your symptoms

Would you like that?

# Symptoms and case presentation:

## Does HARD FLACCID SYNDROME affect my sexual and reproductive health?

Hard Flaccid Syndrome (HFS) can **affect sexual function**, but there is **no evidence that it causes permanent reproductive harm**. Here’s a clear breakdown:

**🔹 Sexual Health**

HFS may affect sexual function in several ways, mainly because of pelvic floor dysfunction, altered penile blood flow, and heightened anxiety or sympathetic nervous system activity.

**Common sexual symptoms related to HFS**

- Difficulty achieving or maintaining erections
- Reduced erection firmness
- Decreased penile sensitivity
- Delayed or difficulty reaching orgasm
- Occasional penile pain or discomfort during arousal

These effects are typically **functional**, meaning they relate to muscle tension, blood flow regulation, and anxiety—not structural damage.

**Important:**
These changes are **reversible** in most individuals as pelvic floor tone normalizes, blood flow stabilizes, and anxiety decreases.

**🔹 Reproductive Health**

There is **no evidence** that HFS directly:

- Damages testicular function
- Affects sperm production
- Reduces fertility
- Causes hormonal imbalance

However, **secondary factors** may influence sexual activity and, indirectly, one’s chances of conceiving:

- Erectile dysfunction making intercourse difficult
- Reduced libido due to chronic discomfort or anxiety
- Ejaculatory difficulty

These do **not** equate to clinical infertility—they are obstacles, not permanent physiological impairments.

**🔹 When to Seek Medical Support**

Seek evaluation from a urologist or men’s pelvic floor specialist if you experience:

- Persistent erectile dysfunction
- New or worsening penile pain
- Ejaculatory pain or difficulty
- Concerns about fertility
- High anxiety or distress related to symptoms

**🔹 Outlook**

The sexual symptoms of HFS are *real* and can be distressing, but with proper therapy (pelvic floor physiotherapy, nervous system regulation strategies, sometimes medical treatment), most patients experience **significant improvement or full recovery**.

**Reproductive health is not damaged** by HFS itself.

If you want, I can also explain:

- Why HFS causes sexual symptoms
- Which sexual symptoms are mechanical vs neurological
- What treatments improve sexual function the fastest
- Whether your specific symptoms fit the typical pattern

## What is the cause of the symptoms of HARD FLACCID SYNDROME?

Hard Flaccid Syndrome (HFS) isn’t fully understood yet, but most experts and clinical reports point toward **a combination of vascular, muscular, and neurological factors**—usually triggered by an injury or strain in the pelvic region. Here’s the most up-to-date explanation of **what likely causes the symptoms**:

**✅ Main Theoretical Causes of HFS Symptoms**

**1. Pelvic Floor Muscle Dysfunction (Hypertonicity / Spasm)**

This is considered the *central mechanism*.
When the pelvic floor becomes **chronically tight**, especially after injury or overstimulation, it can:

- Compress nerves and blood vessels
- Restrict venous outflow and inflow
- Cause a “hard but flaccid” penile state
- Produce burning, pressure, coldness, or numbness sensations

Hypertonic pelvic muscles (especially the **ischiocavernosus, bulbospongiosus, pubococcygeus**) can remain constantly contracted, disrupting normal penile blood flow.

**2. Neurovascular Compression / Irritation**

Several nerves related to sexual function may become irritated, including:

- **Pudendal nerve**
- **Dorsal penile nerve**
- **Perineal branches**

Irritation may occur from:

- Micro-trauma
- Overly tight pelvic muscles
- Prolonged sitting
- Inflammation
- Post-masturbation or sexual activity strain

**Symptoms explained by this:**

- Tingling or burning in the penis
- Hypersensitivity or numbness
- Pelvic pain
- Erectile difficulties
- Hard, semi-engorged flaccid state

**3. Venous Outflow Dysfunction**

A commonly described mechanism is **impaired venous drainage** from the penis due to:

- Pelvic floor muscle compression
- Engorged fascia
- Decreased relaxation in penile smooth muscle

This leads to:

- A semi-firm “hard flaccid”
- A feeling of blood being “trapped”
- Cold or full sensations
- Reduced spontaneous erections

This is *not* the same as venous leak erectile dysfunction but can mimic some symptoms temporarily.

**4. Autonomic Nervous System Dysregulation**

Chronic stress, anxiety, or a sudden traumatic sexual event can dysregulate the autonomic nervous system.

This can push the pelvis into a **sympathetic overdrive (“fight or flight”) state**, producing:

- Constant pelvic muscle tension
- Reduced blood flow to the penis
- Anxiety-pain feedback loops
- Persistent symptoms even after the original injury heals

**5. Micro-Trauma or Overuse Injury**

A common trigger reported by patients is:

- Intense or prolonged sexual activity
- Aggressive masturbation
- Je**king with numbing creams or tight grip
- Sudden bending injury
- Excessive Kegel exercises

This may cause:

- Minor penile tissue injury
- Local inflammation
- Tightening of surrounding muscles
- Nerve sensitization

Even when the injury heals, the pelvic floor may stay hypertonic, maintaining symptoms.

**🔍 Putting It Together: What Actually Causes the Symptoms?**

HFS symptoms are believed to come from a cycle:

1. **Triggering event** → microtrauma, overuse, strain, or anxiety
2. **Pelvic floor tightens** as a protective reflex
3. **Nerves and blood vessels get compressed**
4. **Penile blood flow becomes abnormal** → hard flaccid state
5. **Symptoms cause anxiety** → pelvic floor tightens more
6. Cycle continues

This model explains **every common symptom**:
✔ hard flaccid penis
✔ reduced sensitivity
✔ erectile changes
✔ pelvic floor pain
✔ tingling, coldness
✔ symptoms worsening when standing, stress, or sexual activity
✔ improvement with relaxation, stretching, and pelvic therapy

**⚠️ Important Note**

HFS is considered a **functional condition**, not a structural one.
Most men recover or significantly improve with:

- Pelvic floor physical therapy
- Relaxation training
- Nerve desensitization
- Lifestyle changes
- Addressing anxiety or stress cycles

It **does not cause structural damage**, infertility, or permanent disability.

If you want, I can also explain:
🔸 the most common triggers of HFS
🔸 which symptoms are dangerous and which are not
🔸 how to differentiate HFS from other conditions
🔸 how each treatment works and why

Just let me know.

## Are there long term side effects of HARD FLACCID SYNDROME?

**Short answer:**
Hard Flaccid Syndrome (HFS) is not known to cause *permanent physical damage* to sexual organs, fertility, or long-term reproductive function. However, it can lead to long-term **functional**, **psychological**, and **quality-of-life–related** effects if symptoms persist or if it is not managed effectively.

Below is a clear, evidence-informed breakdown.

**✅ Long-Term Effects and Side Effects of Hard Flaccid Syndrome**

**1. Persistent Erectile and Sexual Function Issues (Functional, Not Structural)**

If HFS persists for months or years, some individuals report:

- Ongoing difficulties achieving or maintaining erections
- Reduced sensitivity
- Flaccid firmness/fullness
- Difficulty relaxing pelvic floor muscles
  **Important:** These are considered *functional* rather than *structural* issues. No studies show permanent penile tissue damage from HFS.

**2. Chronic Pelvic Floor Dysfunction**

Many long-term cases involve:

- Pelvic floor hypertonicity
- Trigger points
- Urinary urgency or weak flow
- Perineal or penile discomfort

This **can become chronic** if not treated but is generally reversible with proper therapy.

**3. Psychological / Mental Health Effects**

This is one of the most significant long-term impacts:

- Anxiety (especially sexual performance anxiety)
- Depression
- Health-focused hypervigilance
- Avoidance of sexual activity
- Reduced libido
- Obsessive symptom monitoring

These psychological effects often become *self-reinforcing*, worsening pelvic floor tension and symptoms.

**4. Impact on Relationships & Quality of Life**

Long-term HFS can lead to:

- Reduced sexual confidence
- Relationship stress
- Avoidance of dating or intimacy
- Social withdrawal
  These are common in chronic pelvic pain and sexual dysfunction conditions.

**5. Long-Term Effects of Incorrect or Excessive Treatments**

Some people worsen symptoms due to:

- Overstretching
- Excessive Kegels (worsens tightness)
- Unqualified trigger-point release
- Misuse of devices (penis pumps, traction, etc.)
- Unsupervised medication use

These can create long-lasting pelvic floor or nerve irritation.

**❌ What HFS *Does Not* Cause Long-Term**

Even in chronic cases, current evidence does **not** show that HFS causes:

- Permanent erectile tissue damage
- Infertility
- Testicular damage
- Hormonal imbalances
- Penile anatomical deformity
- Penile shrinkage (any shrinkage is due to muscular tension and reversible)

**🧭 What Reduces the Risk of Long-Term Problems**

If you’re concerned about long-term effects, the following significantly improves prognosis:

- Pelvic floor physiotherapy (relaxation-based)
- Breathwork and parasympathetic rebalancing
- Identifying triggers (porn, stress, pelvic tension, edging, overstimulation)
- Psychosexual therapy if anxiety is major
- Avoiding traumatic or aggressive treatments

Most long-term sufferers improve when symptoms are approached holistically.

**✔️ If you want, I can also explain:**

- Whether HFS can become permanent
- What percentage of patients recover
- Why the condition becomes chronic for some
- A realistic long-term recovery timeline
- Which treatments have the strongest evidence

Just let me know—happy to help.

## Are the symptoms of HARD FLACCID SYNDROME dangerous? Can they be a sign of something worrisome?

Hard Flaccid Syndrome (HFS) can feel alarming because the symptoms involve erections, genital sensation, urination, and pelvic function — but **the symptoms themselves are *not typically dangerous***. However, they **can be a sign that something else is going on**, and they should be taken seriously because they affect quality of life, mental health, and pelvic function.

Below is a clear breakdown so you know what *is* concerning, what *is not*, and when to seek medical evaluation.

**✅ Are the symptoms of HFS dangerous?**

In most cases **NO**, they are not dangerous in the medical-emergency sense.

HFS is usually associated with:

- Pelvic floor muscle dysfunction
- Neurovascular irritation (often pudendal nerve–related)
- Muscular hypertonicity
- Stress-related autonomic dysregulation

These **do not typically progress to permanent damage**, organ failure, or life-threatening issues.

People often fear:

- Erectile tissue damage
- Penile fibrosis
- Loss of fertility
- Testicular dysfunction

➡️ **There is no evidence that HFS causes any of these.**

**⚠️ Can HFS symptoms be a sign of something worrisome?**

**Sometimes**, because similar symptoms can also occur in other conditions. This doesn’t mean HFS is dangerous, but it does mean a medical evaluation is valuable.

HFS-like symptoms may overlap with:

**1. Pelvic floor hypertonicity or spasm (most common underlying factor)**

Not dangerous but can be painful and persistent.
Treatable with pelvic floor physiotherapy.

**2. Pudendal nerve irritation/entrapment**

Causes:

- Numbness or burning
- Perineal heaviness
- Changes in erection quality

Usually managed conservatively; rarely dangerous.

**3. Vascular compression or congestion**

Can cause:

- Engorged but soft penis
- Dysregulated blood flow

Not harmful long-term but uncomfortable.

**4. Post-masturbation strain or microtrauma**

Very common trigger; not dangerous.

**5. Psychological or autonomic dysregulation**

Stress can magnify symptoms but does not indicate permanent damage.

**Situations where symptoms might indicate a different condition:**

These are **rare**, but worth checking if symptoms don’t match typical HFS:

- **Peyronie’s disease** (plaques, bending, palpable lumps)
- **Chronic prostatitis / CPPS**
- **Neurologic disorders** (significant numbness, leg symptoms)
- **Vascular disease** (signs of ischemia—again rare)
- **Urinary issues that worsen over time**

If any of these apply, a urologist evaluation is important.

**🚨 Red flags to watch for**

If you experience ANY of the following, the issue may be *beyond* HFS:

- Sudden, severe penile pain with discoloration
- Progressive or total loss of genital sensation
- Difficulty urinating or urinary retention
- Visible deformity of the penis
- Severe pain radiating down the legs
- Unexplained fever or systemic illness

These are not typical of HFS and should be checked promptly.

**✔️ The good news**

Most people with HFS do **not** have anything medically dangerous.
The condition:

- Is uncomfortable
- Is anxiety-provoking
- Interrupts sexual function
- But **does not usually cause permanent damage or infertility**.

And importantly:
**A significant number of people improve with proper pelvic floor therapy, lifestyle changes, and managing triggers.**

**🙋‍♂️ If you want, I can also explain:**

- What specifically causes each symptom
- How to tell if your symptoms fit typical HFS
- What tests a urologist usually performs
- What recovery timelines look like

Just let me know which direction you want to explore.
